# Functionalization of the Chalcone Scaffold for the Discovery of Novel Lead Compounds Targeting Fungal Infections

**DOI:** 10.3390/molecules24020372

**Published:** 2019-01-21

**Authors:** Francesca Bonvicini, Giovanna A. Gentilomi, Francesca Bressan, Silvia Gobbi, Angela Rampa, Alessandra Bisi, Federica Belluti

**Affiliations:** 1 Department of Pharmacy and Biotechnology, Alma Mater Studiorum-University of Bologna, Via Massarenti 9, 40138 Bologna, Italy; francesca.bonvicini4@unibo.it (F.B.); giovanna.gentilomi@unibo.it (G.A.G.); 2 Unit of Microbiology, Alma Mater Studiorum-University of Bologna, S. Orsola-Malpighi Hospital, Via Massarenti 9, 40138 Bologna, Italy; 3 Department of Pharmacy and Biotechnology, Alma Mater Studiorum-University of Bologna, Via Belmeloro 6, 40126 Bologna, Italy; francesca.bressan@studio.unibo.it (F.B.); silvia.gobbi@unibo.it (S.G.); angela.rampa@unibo.it (A.R.); alessandra.bisi@unibo.it (A.B.)

**Keywords:** anti-virulence agents, biofilm, *Candida albicans* chalcone scaffold, clinically relevant yeasts, fluorine atom, yeast-to-hyphae transition

## Abstract

The occurrence of invasive fungal infections represents a substantial threat to human health that is particularly serious in immunocompromised patients. The limited number of antifungal agents, devoid of unwanted toxic effects, has resulted in an increased demand for new drugs. Herein, the chalcone framework was functionalized to develop new antifungal agents able to interfere with cell growth and with the infection process. Thus, a small library of chalcone-based analogues was evaluated in vitro against *C. albicans* ATCC 10231 and a number of compounds strongly inhibited yeast growth at non-cytotoxic concentrations. Among these, **5** and **7** interfered with the expression of two key virulence factors in *C. albicans* pathogenesis, namely, hyphae and biofilm formation, while **28** emerged as a potent and broad spectrum antifungal agent, enabling the inhibition of the tested *Candida* spp. and non-*Candida* species. Indeed, these compounds combine two modes of action by selectively interfering with growth and, as an added value, weakening microbial virulence. Overall, these compounds could be regarded as promising antifungal candidates worthy of deeper investigation. They also provide a chemical platform through which to perform an optimization process, addressed at improving potency and correcting liabilities.

## 1. Introduction

Life-threatening invasive fungal infections (IFIs) have been widely recognized as the “hidden killers” of immunocompromised individuals such as patients subjected to organ transplantation and those affected by cancer or AIDS [1,2]. Recently, IFIs have become a severe health problem due to their association with high incidence and mortality [3]. Candidiasis is the main etiologic factor triggering IFIs, with a mortality rate ranging from 46% to 75% for *Candida albicans*, and from 20% to 70% for *Cryptococcus neoformans* [2]. 

*Candida* spp. are members of the commensal human microflora in anatomically distinct sites (oral, gastrointestinal, and genitourinary tracts) but, as stated above, under an extensive variety of circumstances (specific immune defects, mucosal or cutaneous barrier disruption, ageing, diabetes, AIDS) they can be responsible for a wide spectrum of serious clinical symptoms. The pathogenicity of the *Candida* spp. is attributed to critical virulence factors, such as yeast‒hyphal transition, adherence to surfaces, production of proteolytic enzymes, and the formation of biofilm, a structure consisting of multicellular microbial communities embedded in a self-produced polymeric matrix [4]. One of the main consequences of the biofilm mode of growth is the enhanced resistance to host defense mechanisms and antifungal drugs, which is why biofilm-associated infections are frequently refractory to conventional therapy. Even if the incidence of resistance among human fungal pathogens is low to moderate compared to antibiotic resistance among bacterial pathogens, the rising problem of multi-drug resistance is becoming a major concern for clinicians. Moreover, epidemiological data reveal that, although *C. albicans* remains the most common species isolated in superficial and invasive fungal infections, the distribution is changing and other yeasts, including opportunistic and ubiquitous saprophytic fungus, are increasingly recovered in immunocompromised hosts, particularly those with central venous catheters or other indwelling devices [5,6].

**Figure 1:**
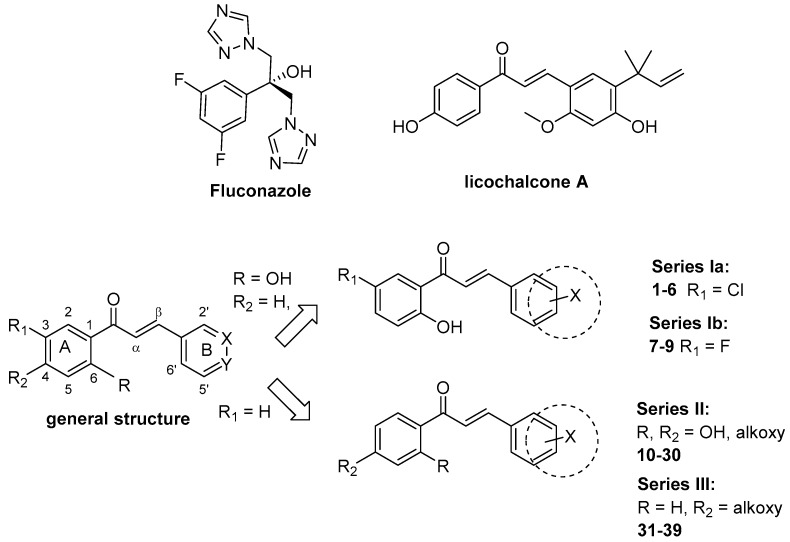
Structure of fluconazole, licochalcone A, and general structures of the tested chalcones.

At present, only a limited number of antifungal agents, namely fluconazole (FLC, Figure 1), voriconazole, and itraconazole, most of which are characterized by an azole function, are in preclinical or clinical studies or available for IFIs treatment [7]. Several limitations such as restricted spectrum of action, toxicity, low efficacy, emergence of resistance, and critical pharmacodynamic and pharmacokinetic profiles, have been recognized to plague this class of therapeutics [8]. On the other hand, Amphotericin B, a very effective and broad-spectrum therapeutic agent for fungal infections, has also shown several side effects [9]. In this scenario, the discovery of novel compounds active against human pathogenic yeasts is an urgent need. Ideally, an antifungal agent should be broad-spectrum, effective against both morphotypes of the yeast (blastospore and hyphae) and biofilms, but also able to reduce the expression of virulence factors. Valuable in this respect are natural products (NPs) and plant-derived secondary metabolites, which exert antimicrobial activity without conferring resistance [10]. NPs, small molecules synthesized by the plant kingdom, define evolutionarily chosen “privileged structures” [11] since they have evolved in a natural selection process to achieve optimal interactions with biological macromolecules. Thus, NPs-inspired compound collections may allow the identification of new molecular templates, suitable for the design of new effective antimicrobial agents. The pharmaceutical industry has extensively relied on NP classes for the discovery of antimicrobics, including polyenes and echinocandins [12]

In this respect, chalcones (1,3-diaryl-2-propen-1-ones) are a prominent class of secondary metabolites and precursors of flavonoids endowed with a wide range of activities, including anti-oxidant, anti-metastatic, anti-inflammatory, and antimicrobial, as extensively reported in many reviews [13]. The molecular basis of chalcones pleiotropic behavior, involving the modulation of multiple biochemical pathways, are likely to be ascribed to their sulfhydryl reactive α,β-unsaturated carbonyl function, a motif largely found in a variety of bioactive NPs. Indeed, the chalcone scaffold demonstrated its suitability to serve as a chemical platform to obtain, upon properly addressed modifications, small compound libraries that may allow performing structure‒activity relationship (SAR) studies [14,15].

Licochalcone A (Figure 1 and Table 1), isolated from the roots of the Chinese plant *Glycyrrhiza glabra*, has been shown to inhibit the in vitro growth of different strains of *C. albicans,* both sensitive and resistant to FLC, with minimum inhibitory concentration (MIC) values in the 62.5–150 μM range. Moreover, at 650 μM concentration, it reduces yeast viability within a mature biofilm [16]. 

## 2. Results

### 2.1. Design Strategy

In an effort to uncover new starting points for antifungal drug discovery, an in-house small library of chalcone-based analogues (Table 1) was screened against *C. albicans,* as a continuation of our exploration of this privileged scaffold as suitable template for developing anti-infective agents. Indeed, some members of the library have been previously tested versus *Leishmania donovani* [17], and a number of compounds endowed with encouraging in vitro activity were identified. To gain insight into the key features for eliciting the antifungal effect, the chemical variability of the chalcones regarded the substitution patterns of the A- and B-rings, as depicted in Figure 1, resulting in Series I, II, and III. Concerning the A-ring: a) Series I was characterized by a 2,5-substutution pattern; in particular, a hydroxyl group was introduced into the 2-position, while a halogen atom such as chlorine or fluorine occupies the 5-position to give Series Ia and Ib, respectively. b) Series II was characterized by a 2,4-disubstitution pattern; in details, position-2 was decorated with hydroxy, methoxy, or propargyloxy functions, while various alkoxy functions were appended into the 4-position. c) Series III contained an alkoxy function into the 4-position. Regarding the B-ring, the following substituents were frequently introduced: 2,4-diCl, 4-dimethylamino, 4-nitro, 4-fluorine, 3- and 4-pyridyl. A number of tested compounds were characterized by a pyridine heterocyclic nucleus (**12**, **13**, **16**, **20**, **21**, **24**, **25**, **32**, **33**, **36**, **37**) as a privileged moiety present in several anti-infective agents [18], and by a F-phenyl ring (**2**–**5**, **7, 8, 10**, **18**, **22**, **28**‒**31**). This small and highly electronegative atom confers special chemical reactivity to the molecule by imparting several favorable properties including improved metabolic stability, selectivity, and efficacy in binding [19]. Taking advantage of these important issues, we successfully exploited this substituent for drug discovery purposes [20,21]. 

### 2.2. Chemistry

**Scheme 1:**
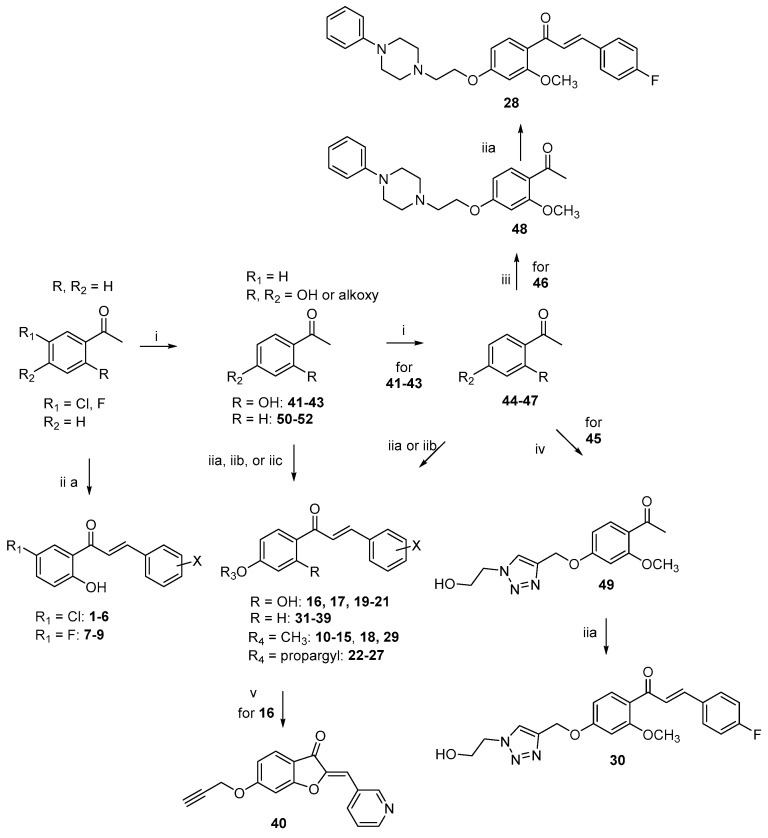
Synthetic route for obtaining analogues 1–40. Reagent and conditions: i) selected alkyl halide, K_2_CO_2_, acetone, reflux; ii) selected aldehyde, EOH, a) Route A: KOH/H_2_O 50%, rt; b) Route B: piperidine/AcOH, reflux, c) Route C: Ba(OH)_2_, rt; iii) phenylpiperazine, TEA, THF, rt; iv) 2-azidoethan-1-ol, CuSO_4_, Na-ascorbate, DMSO; v) NaOH, H_2_O_2_, EtOH, 0 °C-rt.

The final compounds were obtained as described in Scheme 1. The Williamson etherification of 2,4-dihydroxyacetophenone with the appropriate alkyl bromide in the presence of K_2_CO_3_ allowed obtaining the acetophenone-based intermediates (**41**–**45, 46, 47**). In particular, treatment of 2,4-dihydroxyacetophenone with 3,3-dimethylallylbromide, propargylbromide or 1-Br,2-Cl-ethane allowed obtaining **41**, **42** [17] and **43**, respectively [21] that were subsequently alkylated with methyliodide to give **44**, **45** [17], and **46**. Alkylation of **42** with propargylbromide gave **47** [17], which was then reacted with phenylpiperazine to achieve intermediate **48**. Acetophenone **49** was obtained through a Huisgen cycloaddition (click chemistry) reaction between the alkyne **45** and 2-azidoethan-1-ol in the presence of CuSO_4_ and Na‒ascorbate as catalysts. An aldol condensation (Claisen‒Schmidt) procedure allowed obtaining the chalcone-based analogues. In detail, three different reaction conditions were employed: 50% aqueous NaOH solution; piperidine/acetic acid; Ba(OH)_2_ reported as Routes A, B, and C, respectively. 

### 2.3. Biological Evaluation

A screening pipeline was developed to assess the therapeutic potential of the synthesized molecules (**1**–**40**). The antifungal activity was firstly investigated as ability to affect in vitro *C. albicans* growth (control strain, ATCC 10231). A set of compounds were then selected for their ability to show an inhibitory activity superior to 50% at 100 μM, and for these the IC_50_ were determined. Afterwards, these derivatives were subjected to dose‒effect safety experiments in mammalian epithelial cells (Vero, ATCC CCL-81). Chalcones having a positive selectivity index (SI), calculated as CC_50_/IC_50_ ratio, were further investigated to measure their effect on different stages of fungal virulence, namely yeast-to-hyphae morphological transition, filaments invasion, and biofilm formation. Moreover, these active compounds were also assayed on a panel of ten pathogenic yeasts obtained from clinical specimens, including *Candida* spp. and non-*Candida* species, in order to fully characterize their antifungal potential.

#### 2.3.1. Specific Inhibition of *Candida albicans* Growth

The tested analogues **1**–**40** were firstly evaluated against *C. albicans* ATCC 10231 at 100 μM. The commercially available antifungal FLC was used as running control to check the quality of the tests (growth/inhibition of the strains as defined by EUCAST guidelines) [22]. For compounds demonstrating activity against fungal growth superior to 50%, IC_50_ values were determined from a six-point dose‒response curve, obtained in the concentration range 200–6.25 µM. From the data reported in Table 1 (expressed both in µM and in µg/mL) some general structure‒activity relationships (SAR) can be drawn. Positively, sixteen out of the tested forty small molecules successfully inhibited *C. albicans* growth. 

Series Ia provided a number of active derivatives. In detail, the simple phenyl ring (as in compound **1**), along with the 3-F-, 4-F-, and 3,5-di-F-phenyl substitution (compounds **3**, **4**, and **5**, respectively) resulted in potent inhibition of cell growth, with IC_50_ values ranging from 48.9 μM to 57.7 μM. On the contrary, the 2-F-phenyl function negatively affected activity (compound **2**, 35.8% of inhibition), while the 4-dimethylaminophenyl moiety (**6**) resulted in an inactive compound. Replacement of the 5-Cl of the A-ring with a fluorine atom (Series Ib) seemed not to affect the activity of derivative **7**, which maintained a good antifungal effect (IC_50_ = 53 µM), comparable to that of **3**. Surprisingly, a loss of potency, with respect to **4**, was observed for the 4-F substituted **8**, while increased activity was obtained with the 4-dimethylamino (**9**, 38.5% of inhibition).

The effect elicited by the pyridine-based B-ring in conferring antifungal activity was extensively investigated. Among the sub-set with a 4-propargyloxy side chain (compounds **12**, **13**, **16**, **24**, and **25**), promising results were observed (IC_50_ values ranging from 8.1 to 43.8 μM). In particular, **13**, characterized by the methoxy group in the 2-position and the 4-pyridyl function, proved to be one of the most active within the series (IC_50_ = 8.1 μM). The 3-pyridyl analogue **12**, structurally related to **13**, showed decreased inhibitory potency (IC_50_ = 30.7 μM), while compound **16**, the demethylated analogue of **12**, was as active as **13** (IC_50_ value of 10.8 μM). In the same way, the 2,4-di-propargyloxylated **24** and **25** (4- and 3- pyridine, respectively), showed inhibitory values of 26.3 μM and 43.8 μM. Also, analogues **20** and **21**, characterized by the 2-hydroxy and 4-(3,3-dimethylalliloxy) moieties, turned out to remarkably inhibit *C. albicans*, as potencies in the single-digit micromolar range were observed. The pyridine-based chalcones **32**, **33**, **36**, and **37**, in which the 2-substituent was removed (Series III), proved to be devoid of any effect. 

All chalcones bearing the 4-dimethylaminophenyl (**15**, **17**, **27**, **35**, **39**) and the 2,4-di-Cl-phenyl (**11**, **19**, **23**) functions as B–ring were inactive (Series II). Regarding the 4-nitro substitution of the B-ring, good inhibitory effects were observed (**14** and **26**, IC_50_ = 39.2 μM and 36.8 μM, respectively). Again, the corresponding nitro-analogues of Series III (**34** and **38**) turned out to be inactive. 

In addition, the effect elicited by the presence of the fluorine atom on the B-ring was investigated through a number of compounds (**10**, **18**, **22**, **28**–**31**) characterized by different substituents on the A-ring. In this regard, chalcones with simple alkoxylated functions showed poor activity: compound **22** was inactive and analogues **10** and **18** inhibited the *C. albicans* growth with percentages of 23.3 and 34.1, respectively. Interestingly, the decoration of the A-ring with hydrophilic functions (compounds **28**–**31**) showed different behavior. The ethoxyphenylpiperazine (**28**) conferred a good antifungal effect: IC_50_ value of 55.9 μM. On the contrary, **30**, characterized by a 2-hydroxyethyl-1*H*-1,2,3-triazole, proved to be inactive. In addition, poor inhibition was noticed for **29** with a more hydrophilic phenol group. Conversely, **31**, with a 4-hydroxyethoxy side chain, displayed a very good inhibitory effect (IC_50_ = 12.0 μM). Finally, the cyclization of the chalcone scaffold into the constrained 2-arylidenebenzofuran-3-one (aurone), still maintaining the unsaturated carbonyl framework, was performed for **13**, to obtain derivative **40**. This last modification caused a loss in activity, leading us to assume that an optimal inhibitory effect is governed by the presence of the propen-1-one flexible linker between aryl A- and B-rings. 

#### 2.3.2. Cytotoxicity Assay

Fungi and mammalian cells share analogous cellular and biochemical pathways; thus, an ideal antifungal agent should be devoid of unwanted side effects. In light of the liabilities of the chalcone scaffold [23], our sixteen best-performing compounds were tested on mammalian epithelial cells (Vero) treated for 72 h with different concentrations of chalcones (200‒6.25 µM range), and cell viability was evaluated. Table 2 reports CC_50_ values in both µM and in μg/mL. This cytotoxicity assay allows us to establish whether the observed effect is associated with a specific ability to affect fungal growth and not purely linked to a general toxic effect.

In particular, compounds **4**, **5**, **14**, and **28** did not display any toxicity, as they did not interfere at all with Vero metabolism, even at the highest concentration of 200 μM. In addition, compounds **1**, **3**, and **7**, showing triple-digit micromolar CC_50_ values (174.1 µM, 151.8 µM, and 181.7 µM, respectively), only slightly affected eukaryotic cell growth, and could therefore be considered non-toxic.

Analogue **20** was moderately toxic (CC_50_ = 54.3 µM); nevertheless, its good antifungal effect (IC_50_ = 16.8 µM) somewhat counterbalances this drawback and, generally, for this compound, an acceptable host/fungi selectivity profile could be assumed. Altogether, these results point to **20** as a promising compound worth modifying to decrease the toxicity. 

Conversely, compounds **13**, **16**, **21**, **24**–**26**, and **31** were associated with a certain degree of toxicity since they showed CC_50_ values in the micromolar range that were not significantly different from the IC_50_ values on *C. albicans*. Therefore, their activity on yeast growth could be considered a generic cytotoxic effect, thus precluding any further investigation. 

Overall, considering that all compounds belonging to Series I were non-toxic, its key chemical features, namely the 2-hydroxy-5-halo substitution pattern of the A-ring, coupled with fluorine decoration of the B-ring, are expected to lead to effective antifungal agents. 

Moreover, analogues **28** and **31**, both bearing a 4-F aryl ring, showed the opposite behavior. The measured toxic effects could be ascribed to the decoration pattern of the A-ring. This trend is also found for the couple **14**/**31** endowed with a 4-nitrophelyl moiety and differing from the substituent appended at the 2-position (methoxy/propargiloxy). 

Since analogues **1**, **3**–**5**, **7**, **14**, and **28** displayed a good safety profile, they were considered for additional evaluations regarding their antimicrobial profile. Their IC_50_ values are comparable to those measured for the lichochalcone A over different strains of *C. albicans* in a recently published paper [16], again confirming chalcone as a privileged scaffold, suitable for the development of antifungal agents.

The above in vitro toxicity evaluation demonstrated some liabilities of the chalcone scaffold and helped us to gain insight into the chemical features affecting or modulating toxic effects. Of note, the pyridine as B-ring undoubtedly represents an issue that needs to be addressed in designing the next generation of derivatives. 

#### 2.3.3. Inhibition of *Candida* spp. and non-*Candida* Yeasts

After we assessed the selective inhibition of chalcones **1**, **3**–**5**, **7**, **14**, and **28** over *C. albicans* ATCC 10231, we further assayed them towards seven clinical isolates of *Candida* spp. and three non-*Candida* yeasts (Table 3 and Table 4, respectively). The *C. albicans* strain recovered from a clinical specimen displayed a susceptibility profile similar to that defined for the control strain, with some slight differences regarding compounds **3** and **5**. This confirms the need to include in the antifungal screening not only standard strains with a stable, defined phenotype and genotype, but also clinical isolates that are representative of pathogens circulating in the population [24]. Moreover, all the selected compounds showed antifungal activity against *C. lusitaniae*, even if to a different extent, and a good effect was observed for **3**, **4**, **7**, and **28**. On the contrary, compounds **1**, **4**, **14**, and **28** proved to inhibit *C. utilis* growth, with **14** and **28** being the most potent, while only **3** and **28** affected *C. parapsilosis* growth. Among the tested clinical strains, *C. krusei*, *C. glabrata*, and *C. tropicalis* exhibited the most resistant phenotypes, only being susceptible to chalcone **28**. This is a promising result as the two latter non-*Candida* species account for the majority of all the infections not caused by *C. albicans* and their increasing resistance to the azole drug class is of great concern. In addition, chalcone **28** exhibited a similar inhibitory activity compared to FLC for *C. krusei* and against some clinical isolates IC_50_ values were around 25 μM. These values perfectly comply with the criteria suggested by Cos et al. [24] defining the anti-infective potential of a small molecule. 

**Figure 2:**
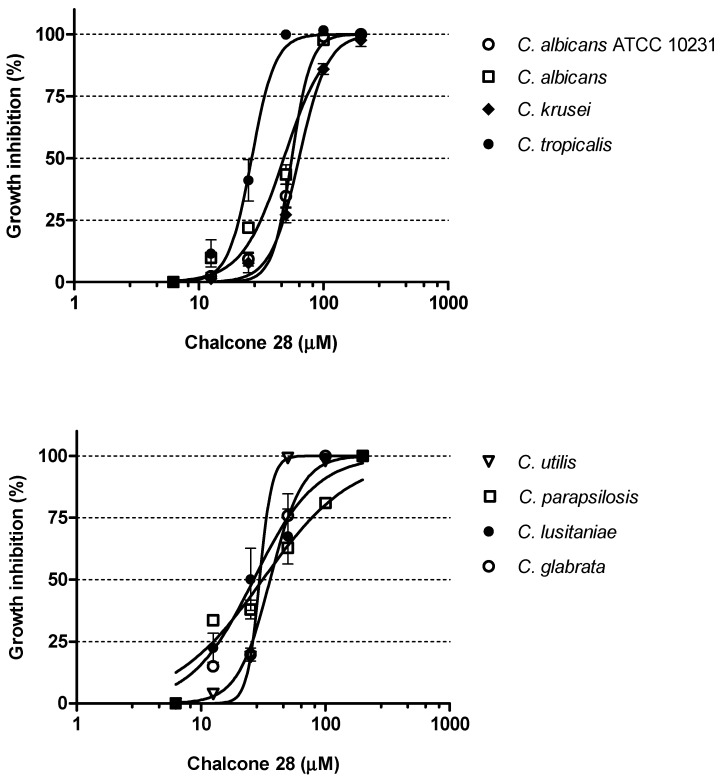
Dose–response curves of chalcone **28** obtained with *C. albicans* ATCC 10231 and all *Candida* spp. clinical isolates.

The potency of chalcone **28** is also demonstrated by the distinctive sigmoidal form of the dose‒response curves (Figure 2 and Table S1). Curves reached 100% of response, indicating its efficacy to completely inhibit yeast growth, and the steep slopes suggested its high effectiveness against the tested strains, as small increases in the doses corresponded to great responses. Just as exceptions, the efficacy of chalcone **28** was slightly lower towards *C. parapsilosis* and *C. lusitaniae* compared to the other clinical isolates, and a shallower curve with a lower slope factor was generated.

Concerning the activity of the seven selected analogues against the other clinically relevant yeasts, they exhibited an overall strong inhibitory effect on *C. neoformans* and *R. mucilaginosa* compared to FLC (Table 4). Chalcones **5**, **7**, and **28** displayed potent two-digit micromolar IC_50_ values on the *C. neoformans* isolate, indicating a potentially relevant clinical impact; indeed, infections by this opportunistic pathogen give rise to most cases of AIDS-related meningitis worldwide, and there has been an increase in the percentage of *Cryptococcus* isolates found to have some degree of FLC resistance. Chalcone **28** exhibited against this pathogen the same potency and efficacy observed for the other tested *Candida* spp. isolates (Figure S1 and Table S2).

#### 2.3.4. Inhibition of Yeast-to-Hyphae Morphological Transition

The ability of the seven chalcones to weaken microbial virulence was further investigated in *C. albicans* ATCC 10231. A key virulence factor expressed by *C. albicans* that significantly contributes to its pathogenicity is the transition of yeast cells to filamentous cells. The formation of germ tube and mycelium is an essential process for host tissue invasion, damage to mucosal epithelia, escape from host immune cells, and blood dissemination. Additionally, the conversion of *C. albicans* from yeast to hyphae is related to the expression of specific adhesins that are involved in the formation of biofilms [4]. For this purpose, *C. albicans* was cultured for 2 h in an RPMI-1640 medium supplemented with serum at 10% and containing the chalcones **1**, **3**–**5**, **7**, **14**, and **28** at IC_50_ values, and then evaluated for their effect on hyphae formation.

**Figure 3:**
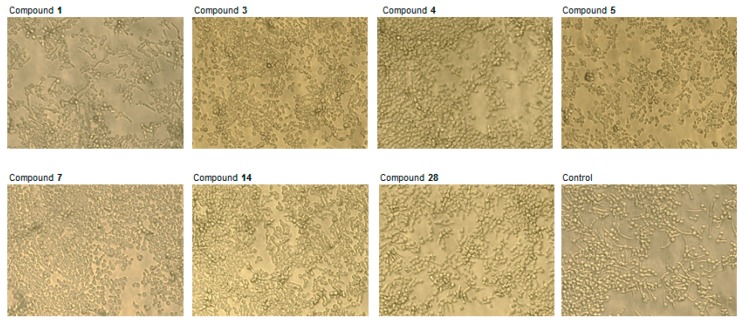
Structural framework of *C. albicans* ATCC 10231 grown for 2 h in RPMI1640-FCS culture medium containing chalcones at IC_50_ values. Visual differences in cell morphotypes can be observed among the positive untreated control and cells at the different experimental conditions. DMSO was used at 0.5% as the solvent control and it did not affect hyphal transition.

As presented in Figure 3, untreated cells exhibited a prevalence of branched filamentous cells, while cells treated with the compounds at IC_50_ values showed different morphological forms with blastospores, budding cells, and filamentous cells. The ranking of yeast-to-hyphae inhibition, as determined by the visual observation of cultures, was **1** < **28** < **14** < **4** < **5** < **3** < **7**. In particular, the *C. albicans* control strain treated with chalcone **7** mainly formed blastospores, some rare budding cells but no hyphae; thus, **7** interfered in vitro with the morphological plasticity of *C. albicans,* which is a crucial virulence determinant for epithelial and endothelial invasion and accession to the bloodstream. 

#### 2.3.5. Inhibition of *C. albicans* Filamentation in Hyphal-Inducing Media

**Figure 4:**
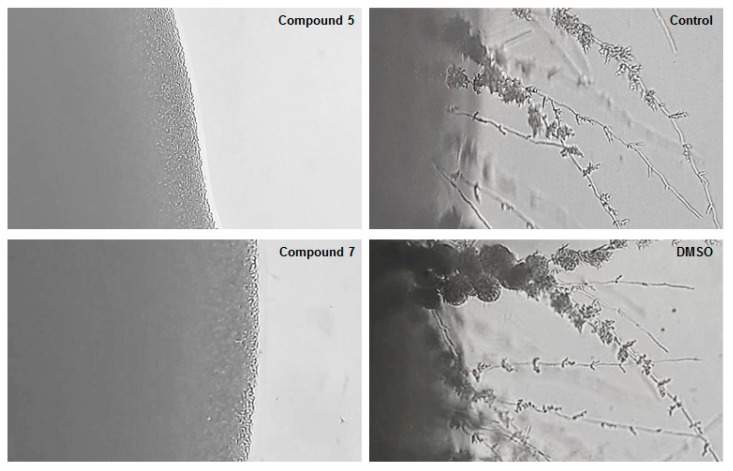
Impact of chalcones **5** and **7** at IC_50_ values on *C. albicans* ATCC 10231 invasion potential determined in the *Spider* agar assay. The colony edges were imaged using a light microscope at the different experimental conditions. DMSO, used as control, did not interfere with *C. albicans* filamentation. The presence of pseudohyphal growth area at the colony edges was determined using a light microscope.

In order to further support the activity of some selected chalcones against virulence attributes of *C. albicans* ATCC 10231 the standard test simulating the filamentation into the tissue (i.e., “Spider” agar assay) was carried out and results are shown in Figure 4. Filaments (hyphae/pseudohyphae) seen under a light microscope at the edge of the colonies in the control medium and in the solvent control were large and formed a broad spider-branched zone around the mycelium. On the contrary, *C. albicans* spotted on the *Spider* medium containing chalcones **5** and **7** at IC_50_ values were unable to penetrate the medium and only blastospores were visualized in the dense mass of the colonies. These data suggested that these compounds inhibit not only the first stage of hyphal growth involving the production of germ tube and hyphae, but also the ability of *C. albicans* to invade a solid medium in the long term.

#### 2.3.6. Antibiofilm Activity

**Figure 5:**
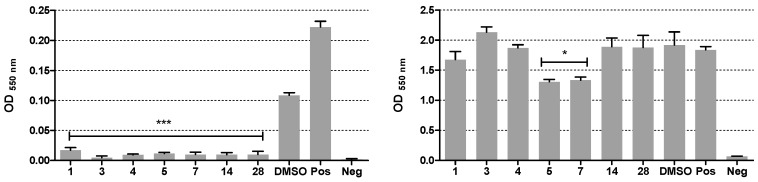
Antibiofilm activity of the selected chalcones on biofilm formation (left panel) and disruption (right panel). Values are reported as mean ± SD. Biofilm formation: *** *p* < 0.0005 for chalcones **1**, **3**–**5**, **7**, **14**, and **28** vs. DMSO/positive control. Biofilm disruption: * *p* < 0.05 for chalcones **5** and ***7*** vs. DMSO/positive control. The negative control consists of the medium only.

Compelling evidences suggest biofilm formation is very often associated to *C. albicans* infections and is responsible for the therapeutic failure and/or resistance phenomena. Biofilms are complex functional communities of one or more species of microorganisms that are encapsulated in a matrix of extracellular polymeric substances. Their development is caused by the adhesion of the microorganisms community to a solid (inert or living) surface. Indeed, the formed biofilm being a complex matrix represents a serious virulence factor that is difficult to eradicate. This physical barrier hampers the antifungal agent from reaching the pathogens and exerting its activity. The antibiofilm effects of the most active compounds, also able to influence yeast morphogenesis (**1**, **3**–**5**, **7**, **14**, and **28**), were investigated in vitro on *C. albicans* ATCC 10231, evaluating their ability to inhibit the biofilm formation process and/or disrupt a mature biofilm. In details, *C. albicans* cells were incubated in the presence of these compounds at IC_50_ values and, following a 24-h incubation, the biofilm mass was quantitatively evaluated by crystal violet staining and destaining solution that was measured spectrophotometrically. As reported in Figure 5, even if the solvent contributed to the inhibition, all tested compounds were able to effectively reduce the formation of the biofilm mass with the following order of potency: **3** > **4** ~ **7** ~ **5** ~ **14** ~ **28** > **1**. This effect could be ascribed to the change in cell wall components that is critical for *C. albicans* adhesion to surfaces; indeed, chalcones may act towards fungal cells by interfering with different biosynthesis, all contributing to disrupt the fungal cell wall [25].

In parallel, the selected compounds were added to a preformed mature biofilm of *C. albicans* and their effect was expressed as the reduction of the biofilm metabolic activity. The data shown in Figure 5 demonstrated that only two molecules (**5** and **7**) slightly diminished the metabolism of sessile cells, at a statistically significant level, even if to a minimal extent (<25%). As currently available antifungals have marginal effect against biofilms [26], the activities of these chalcones are therefore of relevance, and new therapeutic agents targeting biofilms are desperately needed, mainly for the treatment of persistent device-associated infections. Interestingly, chalcone **7** displayed remarkable antifungal activity, specifically against *C. albicans*, affecting several virulence factors: yeast growth, germ tube formation, and biofilm production. In addition, among the seven active compounds, it exhibited the greatest inhibitory activity against the clinical isolate of *C. albicans* (IC_50_ = 32.1 μM). 

## 3. Discussion

A small library of 40 chalcone-based analogues was evaluated for their antifungal potential. The study delivered three lead molecules (**28**, **5**, and **7**) endowed with relevant antifungal features. Analogue **28** proved to be the most interesting since it exhibited a broad spectrum of activity, being able to effectively inhibit several *Candida* spp. such as *C. albicans, C. parapsilosis, C. lusitaniae, C. krusei, C. tropicalis, C. glabrata*, and *C. utilis,* and a broad array of clinically relevant yeasts, including *C. neoformans*, which is responsible of one of the most fatal meningoencephalitis in AIDS patients across the globe. The concomitant presence of the phenylpiperazine, protonable at physiological pH, and the 4-F-phenyl moiety could be responsible for the good antifungal effect of this chalcone. 

The antifungal effect of compounds **5** and **7** could mainly be ascribed to an antivirulence effect; as they weakened hyphae and biofilm production, factors strongly involved in *C. albicans* pathogenesis, this behavior perfectly meets the current paradigm of anti-infective therapeutics [27]. The presence of a halogen (chlorine or fluorine atom) in the 5-position of the chalcone A-ring seems to be a favorable substitution pattern. 

Overall, the pyridine heterocycle as B-ring demonstrated to confer an impressive ability to inhibit *C. albicans* growth; unfortunately, this effect is likely due to the intrinsically toxic effects of the molecules. However, this last sub-set represents a promising starting point in optimization efforts. In fact, we anticipate that it will be possible to overcome the current drawbacks by proper functionalization of the heterocyclic nitrogen atom. 

## 4. Experimental Section

### 4.1. Chemistry

Starting materials, unless otherwise specified, were used as high-grade commercial products. Solvents were of analytical grade. Reaction progress was followed by thin-layer chromatography (TLC) on precoated silica gel plates (Merck Silica Gel 60 F254, Darmstadt, Germany) and then visualized with a UV 254 lamplight. Chromatographic separations were performed on Merck silica gel columns by the flash method (Kieselgel 40, 0.040–0.063 mm, Merck, Darmstadt, Germany). Melting points were determined in open glass capillaries, using a Büchi SMP-20 apparatus (Büchi Italia s.r.l., Cornaredo, Mi, Italy) and are uncorrected. ^1^H-NMR and ^13^C-NMR spectra were recorded on a Varian Gemini spectrometer (Scientific instruments, Palo Alto, CA, USA) 400 MHz and 101 MHz, respectively, and chemical shifts (δ) are reported as parts per million (ppm) values relative to tetramethylsilane (TMS) as internal standard; unless otherwise indicated, CDCl_3_ was used as the solvent. Standard abbreviations indicating spin multiplicities are given as follows: s (singlet), d (doublet), t (triplet), br (broad), q (quartet), or m (multiplet); coupling constants (*J*) are reported in Hertz (Hz). Mass spectra were recorded on a Waters ZQ 4000 apparatus Waters Alliance, San Diego, CA, USA) operating in electrospray mode (ES). Analyses indicated by the symbols of the elements were within ± 0.4% of the theoretical values. Compounds were named relying on the naming algorithm developed by CambridgeSoft Corporation and used in Chem-BioDraw Ultra 14.0 (PerkinElmer Inc., Waltham, MA, USA).

#### 4.1.1. Williamson Reaction: General Procedure

A mixture of the selected acetophenone (1.0 eq), alkyl halide (1.0 or 1.1 eq), K_2_CO_3_ (1.0 eq) in acetone was heated under reflux for 6–10 h; reaction progress was monitored by TLC. Upon reaction completion, the mixture was hot filtered and the solvent was evaporated under reduced pressure. The resulting crude product was purified by column chromatography using a mixture of petroleum ether (PE)/ethyl acetate (EtOAc) as eluent to give the desired pure product. 

*1-(4-(2-chloroethoxy)-2-hydroxyphenyl)ethan-1-one* (**46**). Reaction of 2,4-dihydroxyacetophenone (0.76 g, 5.0 mmol) and 1-bromo-2-chloroethane (0.94 g, 0.45 mL, 5.0 mmol) gave a crude product that was purified by column chromatography (PE/EtOAc 4:1) producing **46** as a solid (0.55 g), 85% yield. ^1^H-NMR δ 2.60 (s, 3H, COCH_3_), 3.83 (t, 2H, *J* = 6.0 Hz, CH_2_Cl), 4.26 (t, 2H, *J* = 6.0 Hz, OCH_2_), 6.40 (d, *J* = 2.2 Hz, 1H, H-3) 6.48 (dd, *J* = 8.0 and 2.2 Hz, 1H, rH-5), 7.65 (d, 1H, *J* = 9.2 Hz, H-6). ^13^ C-NMR δ 26.3, 60.55, 69.21, 103.25, 106.74, 131.00, 165.47, 165.87, 201.88. 

*1-(4-(2-chloroethoxy)-2-methoxyphenyl)ethan-1-one* (**47**). Reaction of **43** (0.18 g, 0.87 mmol) and methyl iodide (0.14 g, 0.06 mL, 0.96 mmol) gave a crude product that was purified by column chromatography (PE/AcOEt 9:1) producing **47** as a transparent oil (0.13 g), 78% yield. ^1^H-NMR δ 2.65 (s, 3H, COCH_3_), 3.85 (t, 2H, *J* = 6.0 Hz, CH_2_Cl), 3.96 (s, 3H, OCH_3_), 4.30 (t, 2H, *J* = 6.0 Hz, OCH_2_), 6.50 (d, *J* = 2.2 Hz, 1H, H-3) 6.58 (dd, *J* = 8.0 and 2.2 Hz, 1H, H-5), 7.67 (d, 1H, *J* = 9.2 Hz, H-6). ^13^ C-NMR δ 26.10, 42.54, 45.97, 75.44, 101.23, 106.78, 119.84, 130.67, 161.74, 164.74, 199.55. 

*1-(4-(2-hydroxyethoxy)phenyl)ethan-1-one* (**50**). Reaction of 4-hydroxyacetophenone (5.0 mmol, 0.90 g) and 2-bromoethanol (5.5 mmol, 0.68 g) gave the crude final product that was purified by column chromatography (PE/EtOAc 4:1) to produce **50** as a white solid (0.63 g), 75% yield. ^1^H-NMR (acetone d_6_) δ 2.58 (s, 3H, CH_3_), 3.92–3.98 (m, 2H, CH_2_OH), 4.07 (t, *J* = 4.4 Hz, 1H, OH), 4.15 (t, *J* = 6.4 Hz, 2H, OCH_2_), 6.96 (d, *J* = 8.6 Hz, 2H, H-3 and H-5), 7.96 (d, *J* = 8.8 Hz, 2H, H-2 and H-6). ^13^ H-NMR δ 26.88, 64.22, 69.15, 127.42, 128.75, 129.12, 129.66, 166.84, 198.05.

*1-(4-((3-methylbut-2-en-1-yl)oxy)phenyl)ethan-1-one* (**51**). Reaction of 4-hydroxyacetophenone (0.76 g, 5.0 mmol) and 3,3-dimethylallylbromide (0.82 g, 5.5 mmol) gave a crude final product that was purified by column chromatography (PE/EtOAc 9:1) to produce **51** as a transparent oil (0.97 g), 95% yield. ^1^H-NMR δ 1.78 (s, 3H, CH_3_), 1.79 (s, 3H, CH_3_), 2.54 (s, 3H, COCH_3_), 4.57 (d, *J* = 6.8 Hz, 2H, OCH_2_), 5.47 (t, *J* = 6.8 Hz, 1H, =CH), 6.92 (d, *J* = 8.8 Hz, 2H, H-3 and H-5), 7.92 (d, *J* = 7.2 H-2 and H-6). ^13^C-NMR δ 18.1, 25.7, 26.1, 64.9, 114.2, 118.9, 130.1, 130.4, 138.7, 162.7, 196.6. ^13^ C-NMR δ 18.88, 26.55, 64.58, 114.47, 114.97, 12.032, 129.16, 129.74, 130.54, 138.17, 162.28, 198.27.

*1-(4-(prop-2-yn-1-yloxy)phenyl)ethan-1-one* (**52**). Reaction of 4-hydroxyacetophenone (0.76 g, 5.0 mmol) and propargyl bromide solution 80 wt % in toluene (0.80 g, 5.5 mmol) gave the crude final product that was purified by column chromatography (PE/EtOAc 9:1) to produce **52** as a solid (0.90 g), 93% yield, mp: 73–74 °C. ^1^H-NMR δ 2.54-2.56 (m, 4H, CH and COCH_3_), 4.76 (d, *J* = 4.2 Hz, 2H, OCH_2_), 7.01 (dd, *J* = 2.0 and 8.0 Hz, 2H, H-3 and H-5), 6.95 (dd, *J* = 2.0 and 8.0 Hz, 2H, H-2 and H-6). ^13^ C-NMR δ 26.33, 56.47. 78.91, 79.32, 114.21, 114.51, 129.06, 129.48, 162.47, 198.41.

*1-(2-Methoxy-4-(2-(4-phenylpiperazin-1-yl)ethoxy)phenyl)ethan-1-one* (**48**). A mixture of **47** (0.26 g, 1.0 mmol), phenylpiperazine (0.24 g, 1.5 mmol) and TEA (0.21 mL, 1.5 mmol) in THF was stirred at rt for 18 h. The solvent was removed by evaporation under reduced pressure and the crude was purified by column chromatography (DCM/MeOH/NH_4_OH 9.5:0.45:0.05) to give **48** as a dense oil (0.30 g), 87% yield; hydrochloride salt mp 131-132 °C. ^1^H-NMR δ 2.50 (s, 3H, COCH_3_), 2.72-2.76 (m, 4H, piperazine), 2.90 (t, *J* = 5.6 Hz, 2H, NCH_2_), 3.22-3.25 (m, 4H, piperazine), 4.19 (t, *J* = 5.6 Hz, 2H, OCH_2_), 6.45 (d, *J* = 1.2 Hz, 1H, H-3), 6.48 (dd, *J* = 8.4 and 1.2 Hz, 1H, H-5), 6.89 (t, *J* = 8.4 Hz, 1H, H-4′), 6.94 (d, *J* = 8.4 Hz, 2H, H-2′ and H-6′), 7.24-7.26 (m, 2H, H-3′ and H-5′), 7.64 (d, *J* = 8.4 Hz, 1H, H-6), 12.7 (s, 1H, OH). ^13^ C-NMR δ 26.53, 49.27, 53.81, 57.03, 56.11, 66. 48, 101.67, 108.21, 114.19, 116.25, 119.95, 129.27, 132.45, 151.40, 195.36.

*1-(4-((1-(2-hydroxyethyl)-1H-1,2,3-triazol-4-yl)methoxy)-2-methoxyphenyl)ethan-1-one* (**49**). A mixture of **45** (0.15 g, 0.73 mmol), 2-azidoethan-1-ol (0.08 g, 0.95 mmol), CuSO_4_ (0.02 g), Na-ascorbate (0.07 g, 0.37 mmol) in DMSO (6.0 mL) was stirred at room temperature for 8 h. Then the mixture was poured in H_2_O and extracted with EtOAc (12 mL × 3). The combined organic layers were dried over Na_2_SO_4_, filtered and the solvent was removed under reduced pressure. The crude was purified by column chromatography (PE/AcOEt 7:3) producing **49** as an oily product (0.19 g), 98% yield. ^1^H-NMR δ 2.61 (s, 3H, COCH_3_), 2.95 (br, 1H, OH), 3.87 (s, 3H, OCH_3_), 4.07 (t, *J* = 5.2 Hz, 2H, NCH_2_), 4.51 (t, *J* = 5.2 Hz, 2H, OCH_2_), 5.23 (s, 2H, OCH_2_), 6.56 (d, *J* = 2.4 Hz, 1H, H-3), 6.60 (dd, *J* = 2.4 and 7.6 Hz, 1H, H-5), 7.78 (s, 1H, triazole CH), 7.79 (d, *J* = 8.0 Hz, 1H, H-6). ^13^ C-NMR δ 26.21, 54.57, 55.08, 60.01, 72.94, 101.24, 106.74, 119.84, 128.45, 131.19, 162.32, 166.82. 194.21.

#### 4.1.2. Claisen–Schmidt Reaction: General Procedure

*Route A.* To a solution of the selected acetophenone (1.0 eq) and aldehyde (1.1 eq) in EtOH (10 mL) a KOH aqueous solution (50% p/v, 1 mL) was added dropwise and reaction mixture was stirred overnight at rt, diluted with H_2_O, and neutralized with aqueous 6N HCl. The formed solid/semisolid product was collected by vacuum filtration or extracted with a suitable solvent. The crude of reaction was then purified by column chromatography or by crystallization.

*Route B*. To a solution the selected acetophenone (1.0 eq) and aldehyde (1.1 eq) in EtOH (10 mL), a solution of piperidine (0.5 mL) and acetic acid (0.3 mL) in EtOH (1 mL) was added. The reaction mixture was heated under reflux for 12–18 h and the reaction progress was monitored by TLC. The solvent was removed under reduced pressure and the crude of reaction was purified by column chromatography or crystallization.

*Route C*. A mixture the selected acetophenone (1.0 eq) and aldehyde (1.1 eq) in EtOH (10 mL), Ba(OH)_2_ (3.3 eq) was added. The reaction mixture was stirred at rt overnight; then diluted with H_2_O and neutralized with aqueous 6N HCl. The formed solid/semisolid product was collected by vacuum filtration or extracted with a suitable solvent. The crude of reaction was then purified by column chromatography or by crystallization.

*(E)-1-(5-Chloro-2-hydroxyphenyl)-3-phenylprop-2-en-1-one* (**1**). Following route A, and starting from 5′-chloro-2′-hydroxyacetophenone (0.17 g, 1.0 mmol) and benzaldehyde (0.12 g, 1.1 mmol) a solid product was obtained that was collected by vacuum filtration. The crude of reaction was purified by crystallization from EtOH producing **1** as a yellowish solid (0.22 g), 85% yield, mp 97–98 °C. ^1^H-NMR δ 6.98 (d, *J* = 8.4 Hz, 1H, H-3), 7.43 (dd, *J* = 1.2 and 8.4 Hz, 2H), 7.33–7.38 (m, 2H, H-3′-H5′), 7-50–7.54 (m, 2H, H-2′ and H-6′), 7.60 (d, *J* = 16.0 Hz, 1H, β-CH=), 7.86 (1H, d, *J* = 1.2 Hz, H-6), 8.04 (d, *J* = 15.6 Hz, 1H, α-CH=), 12.55 (br, 1H, OH). ^13^C-NMR δ 119.21, 124.45, 127.21, 128.75, 128.77, 130.76, 131.32, 137.14, 145.16, 161.08, 192.11. ESI-MS (*m/z*) 259 (M + 1). HRMS ESI + [M + 1]: calcd for C_15_H_11_ClO_2_, 259.0526. Found: 259.0528. 

*(E)-1-(5-Chloro-2-hydroxyphenyl)-3-(2-fluorophenyl)prop-2-en-1-one* (**2**). Following route A, and starting from 5′-chloro-2′-hydroxyacetophenone (0.17 g, 1.0 mmol) and 2-fluorobenzaldehyde (0.14 g, 1.1 mmol), a solid product was obtained that was collected by vacuum filtration and crystallized from EtOH, producing **2** as a white solid (0.13 g), 47% yield, mp 86–87 °C. ^1^H-NMR δ 7.00 (d, *J* = 8.8 Hz, 1H, H-3), 7.15–7.20 (m, 1H, Ar), 7.46–7.48 (m, 2H, Ar), 7.67–7.72 (m, 2H, Ar), 7.70 (d, *J* = 16.0 Hz, 1H, β-CH=), 7.76–7.86 (m, 1H, Ar), 8.04 (d, *J* = 15.6 Hz, 1H, α-CH=), 12.67 (br, 1H, OH). ^13^C-NMR δ 115.16, 119.45, 121.21, 123.11, 124.25, 128.27, 128.75, 131.86, 137.32, 145.16, 161.58, 192.31. ESI-MS (*m/z*): 399 (M+23). HRMS ESI + [M + 1]: calcd for C_15_H_10_ClFO_2_, 277.0431. Found: 277.04533. 

*(E)-1-(5-Chloro-2-hydroxyphenyl)-3-(3-fluorophenyl)prop-2-en-1-one* (**3**). Following route A, and starting from 5′-chloro-2′-hydroxyacetophenone (0.17 g, 1.0 mmol) and 3-fluorobenzaldehyde (0.14 g, 1.1 mmol), a solid product was obtained that was collected by vacuum filtration, purified by column chromatography (PE/EtOAc 9.5:0.5), and crystallized from EtOH, producing **3** as a yellowish solid (0.13 g), 46% yield, mp 75–77 °C. ^1^H-NMR δ 7.02 (d, *J* = 8.8 Hz, 1H, H-5), 7.14-7.17 (m, 1H, Ar), 7.37–7.44 (m, 3H, Ar), 7.49 (dd, *J* = 2.4 and 8.8 Hz, 1H, H-4), 7.57 (d, *J* = 15.2 Hz, 1H, β-CH=), 7.87 (d, *J* = 2.4 Hz, 1H, H-6), 7.91 (d, *J* = 15.2 Hz, 1H, α-CH=). ^13^C-NMR δ 113.26, 114.45, 119.91, 123.81, 124.28, 127.77, 130.20, 131.86, 136.22, 161.16, 162.58, 192.51. ESI-MS (*m/z*) 277 (M + 1). HRMS ESI + [M + 1]: calcd for C_15_H_10_ClFO_2_, 277.0431. Found: 277.0432. 

*(E)-1-(5-Chloro-2-hydroxyphenyl)-3-(4-fluorophenyl)prop-2-en-1-one* (**4**). Following route A, and starting from 5′-chloro-2′-hydroxyacetophenone (0.17 g, 1.0 mmol) and 4-fluorobenzaldehyde (0.14 g, 1.1 mmol), a solid product was obtained that was collected by vacuum filtration and crystallized from EtOH, to obtain **4** as a white solid (0.21 g), 75% yield, mp 79–81 °C. ^1^H-NMR δ 6.99 (d, *J* = 8.8 Hz, 1H, H-5), 7.13 (d, *J* = 8.8 Hz, 1H, H-3′), 7.15 (d, *J* = 8.8 Hz, 1H, H-5′), 7.45 (dd, *J* = 2.4 and 8.8 Hz, 1H, H-4) 7.49 (d, *J* = 15.2 Hz, 1H, β-CH=), 7.68 (d, *J* = 8.4 Hz, 1H, H-2′), 7.69 (d, *J* = 8.8 Hz, 1H, H-6′), 7.80 (d, *J* = 2.4 Hz, 1H, H-6), 7.92 (d, *J* = 15.2 Hz, 1H, α-CH=). ^13^C-NMR δ 116.25, 116.47, 119.19, 120.29, 120.54, 123.57, 128.75, 130.85 136.14, 145.16, 162.08, 192.64. ESI-MS (*m/z*) 277 (M + 1). HRMS ESI + [M + 1]: calcd for C_15_H_10_ClFO_2_, 277.0431. Found: 277.0433. 

*(E)-1-(5-Chloro-2-hydroxyphenyl)-3-(3,5-difluorophenyl)prop-2-en-1-one* (**5**). Following route A, and starting from 5′-chloro-2′-hydroxyacetophenone (0.17 g, 1.0 mmol) and 3,5-di-fluorobenzaldehyde (0.16 g, 1.1 mmol), a solid product was obtained that was collected by vacuum filtration and crystallized from EtOH, to obtain **5** as a white solid (0.20 g), 67% yield, mp 102–103 °C. ^1^H-NMR δ 6.92 (t, *J* = 8.4 Hz 1H, H-4′), 7.02 (d, *J* = 9.2 Hz, 1H, H-3), 7.20 (d, *J* = 6.0 Hz, 2H, H-2′ and H-6′), 7.48 (dd, *J* = 2.0 and 8.8 Hz, 1H, H-4), 7.55 (d, *J* = 15.6 Hz, 1H, β-CH=), 7.85 (s, 1H, H-6), 7.83 (d, *J* = 15.6 Hz, 1H, α-CH=), 12.98 (br, 1H, OH). ^13^C-NMR δ 103.25, 109.47, 119.33, 124.29, 127.53, 130.57, 131.75, 137.14, 138.99, 160.68, 192.33. ESI-MS (*m/z*) 295 (M + 1). HRMS ESI + [M + 1]: calcd for C_15_H_9_ClF_2_O_2_, 295.0337. Found: 295.0339.

*(E)-1-(5-chloro-2-hydroxyphenyl)-3-(4-(dimethylamino)phenyl)prop-2-en-1-one* (**6**). Following route B, and starting from 5′-chloro-2′-hydroxyacetophenone (0.17 g, 1.0 mmol) and 4-(dimethylamino)benzaldehyde (0.16 g, 1.1 mmol), a crude of reaction was obtained purified by column chromatography (PE/EtOAc 7:3), and crystallized from EtOH to obtain **6** as a red solid (0.20 g), 67% yield, mp 125–127 °C. ^1^H-NMR δ 3.10 (s, 6H, CH_3_), 6.79 (d, *J* = 8.4 Hz, 2H, H-2′ and H-6′), 6.97 (d, *J* = 9.2 Hz, 1H, H-3), 7.38 (d, *J* = 15.2 Hz, 1H, β-CH=), 7.41 (dd, *J* = 2.4 and 8.8 Hz, 1H, H-4), 7.62 (d, *J* = 8.4 Hz, 2H, H-3′ and H-5′), 7.88 (d, *J* = 2.8 Hz, 1H, H-6), 7.95 (d, *J* = 14.8 Hz, 1H, α-CH=), 13.10 (br, 1H, OH). ^13^C-NMR δ 40.08, 111.77, 113.41, 120.01, 121.03, 122.05, 123.15, 128.52, 131.14, 135.31, 147.58, 152.54, 161.94. ESI-MS (*m/z*) 302 (M + 1). HRMS ESI + [M + 1]: calcd for C_17_H_16_ClNO_2_, 302.0948. Found: 302.0950.

*(E)-1-(5-Fluoro-2-hydroxyphenyl)-3-(3-fluorophenyl)prop-2-en-1-one* (**7**). Following route A, and starting from 5′-fluoro-2′-hydroxyacetophenone (0.15 g, 1.0 mmol) and 3-fluorobenzaldehyde (0.14 g, 1.1 mmol), a solid product was obtained that was collected by vacuum filtration and crystallized from EtOH to obtain **7** as a white solid (0.18 g), 70% yield, mp 92–94 °C. ^1^H-NMR δ 7.00–7.04 (m, 1H, Ar), 7.15–7.19 (m, 1H, Ar), 7.26–7.25 (m, 1H, Ar), 7.37–7.39 (m, 1H, Ar), 7.41–7.45 (m, 2H, Ar), 7.54 (d, *J* = 15.6 Hz, 1H, β-CH=), 7.56–7.29 (m, 1H), 7.90 (d, *J* = 15.2 Hz, 1H, α-CH=). ^13^C-NMR δ 110.22, 114.44, 118.29, 120.29, 123.44, 123.56, 125.57, 130.45, 136.14, 145.76, 159.11, 162.08, 192.67. ESI-MS (*m/z*) 261 (M + 1). HRMS ESI + [M + 1]: calcd for C_15_H_10_F_2_O_2_, 261.0727. Found: 261.0725.

*(E)-1-(5-Fluoro-2-hydroxyphenyl)-3-(4-fluorophenyl)prop-2-en-1-one* (**8**). Following route A, and starting from 5′-fluoro-2′-hydroxyacetophenone (0.15 g, 1.0 mmol) and 4-fluorobenzaldehyde (0.14 g, 1.1 mmol), a semisolid product was obtained that was extracted with DCM (3 × 30 mL). The unified organic layers were dried over anhydrous Na_2_SO_4_ and evaporated to dryness to give a crude that was purified by column chromatography (PE/EtOAc 95:0.5) and then crystallized from EtOAc/*n*-hexane, to produce **8** as a white solid (0.21 g), 80% yield, mp 94–96 °C. ^1^H-NMR δ 7.02 (dd, *J* = 8.8 and 9.6 Hz, 1H, H-3), 7.17 (d, *J* = 8.4 Hz, 1H, H-3′), 7.18 (d, *J* = 8.4 Hz, 1H, H-5′), 7.23-7.26 (m, 1H, H-4), 7.48 (d, *J* = 15.2 Hz, 1H, β-CH=), 7.58 (dd, *J* = 2.4 and 8.8 Hz, 1H, H-6), 7.68 (d, *J* = 8.8 Hz, 1H, H-2′), 7.69 (d, *J* = 8.4 Hz, 1H, H-6′), 7.92 (d, *J* = 15.2 Hz, 1H, α-CH=), 12.55 (s, 1H, OH). ^13^C-NMR δ 116.26, 116.67, 119.21, 120.27, 120.76, 123.69, 128.69, 130.23, 136.44, 159.90, 162.69, 192.44. ESI-MS (*m/z*) 261 (M + 1). HRMS ESI + [M + 1]: calcd for C_15_H_10_F_2_O_2_, 261.0727. Found: 261.0728.

*(E)-3-(4-(Dimethylamino)phenyl)-1-(5-fluoro-2-hydroxyphenyl)prop-2-en-1-one* (**9**). Following route B, and starting from 5′-fluoro-2′-hydroxyacetophenone (0.15 g, 1.0 mmol) and 4-(dimethylamino)benzaldehyde (0.16 g, 1.1 mmol) a crude of reaction was obtained, purified by column chromatography (PE/EtOAc 7:3), and crystallized from EtOH to obtain **9** as a red solid (0.14 g), 50% yield, mp 131–135 °C. ^1^H-NMR δ 3.09 (s, 6H, CH_3_), 6.75 (d, *J* = 9.2 Hz, 2H, H-3′ and H-5′), 6.97 (dd, *J* = 8.8 and 8.8 Hz, 1H, H-3), 7.24 (dd, *J* = 7.6 and 8.8 Hz, 1H, H-4), 7.34 (d, *J* = 15.2 Hz, 1H, β-CH=), 7.57–7.60 (m, 3H, H-6, H-3′, and H-5′), 7.94 (d, *J* = 15.2 Hz, 1H, α-CH=), 12.89 (s, 1H, OH). ^13^C-NMR δ 41.35, 110.41, 117.55, 117.23, 124.78, 125.88, 145.21, 150.61, 159.41, 159.95, 193.57. ESI-MS (*m/z*) 286 (M + 1). HRMS ESI + [M + 1]: calcd for C_17_H_16_FNO_2_, 286.1243. Found: 286.1244.

*(E)-3-(4-fluorophenyl)-1-(2-methoxy-4-(prop-2-yn-1-yloxy)phenyl)prop-2-en-1-one* (**10**). Following route A, and starting from **45** (0.20 g, 1.0 mmol) and 4-fluorobenzaldehyde (0.14 g, 1.1 mmol), a semisolid product was obtained that was extracted with EtOAc (3 × 30 mL). The unified organic layers were dried over anhydrous Na_2_SO_4_ and evaporated to dryness to give a crude that was purified by column chromatography (PE/EtOAc 8:2), to produce **10** as a solid (0.29 g), 94% yield, mp 196–168 °C. ^1^H-NMR δ 2.58 (t, *J* = 2.4 Hz, 1H, CH), 3.92 (s, 3H, OCH_3_), 4.77 (d, *J* = 2.0 Hz, 2H, OCH_2_), 6.61 (d, *J* = 2.0 Hz, 1H, H-3), 6.65 (dd, *J* = 2.4 and 8.8 Hz, 1H, H-5), 7.09 (d, *J* = 8.8 Hz, 1H, H-3′), 7.10 (d, *J* = 8.8 Hz, 1H, H-6′), 7.43 (d, *J* = 16.0 Hz, 1H, β-CH=), 7.50-7.59 (m, 2H, H-2′ and H-6′), 7.65 (d, *J* = 16 Hz, 1H, α-CH=), 7.76 (d, *J* = 8.8 Hz, 1H, H-6). ^13^C-NMR δ 56.21, 76.54, 101.11, 107.45, 115.88, 118.66, 123.67, 130.11, 130.54, 131.87, 145.64, 162.46, 163.78, 188.32. ESI-MS (*m/z*) 311 (M + 1). HRMS ESI + [M + 1]: calcd for C_19_H_15_FO_3_, 311.1083. Found: 311.1085.

*(E)-3-(2,4-dichlorophenyl)-1-(2-methoxy-4-(prop-2-yn-1-yloxy)phenyl)prop-2-en-1-one* (**11**). Following route A, and starting from **45** (0.20 g, 1.0 mmol) and 2,4-dichlorobenzaldehyde (0.19 g, 1.1 mmol) a solid product was obtained that was collected by vacuum filtration and crystallized from EtOH to give **11** as a pale yellow solid (0.29 g), 84% yield, mp 174–75 °C. ^1^H-NMR δ 2.59 (t, *J* = 2.4 Hz, 1H, CH), 3.92 (s, 3H, OCH_3_), 4.78 (s, 2H, OCH_2_), 6.61 (d, *J* = 2.0 Hz, 1H, H-3), 6.66 (dd, *J* = 2.4 and 8.8 Hz, 1H, H-5), 7.28 (dd, *J* = 2.2 and 8.4 Hz 1H, H-5′), 7.45 (d, *J* = 2.2 Hz, 1H, H-3′), 7.47 (d, *J* = 16.0 Hz, 1H, β-CH=), 7.64 (d, *J* = 8.8 Hz, 1H, H-6′), 7.79 (d, *J* = 8.8 Hz, 1H, H-6), 7.98 (d, *J* = 16.0 Hz, 1H, α-CH=). ^13^C-NMR δ 55.81, 56.71, 76.42, 77.66, 101.10, 107.54, 121.56, 122.47, 124.94, 125.98, 127.15, 129.74, 130.45, 1.3.31, 136.60, 161.74, 167.21, 194.11. ESI-MS (*m/z*) 361 (M+1). HRMS ESI + [M + 1]: calcd for C_19_H_14_Cl_2_O_3_, 361.0398. Found: 361.0399.

*(E)-3-(4-(dimethylamino)phenyl)-1-(2-methoxy-4-(prop-2-yn-1-yloxy) phenyl)prop-2-en-1-one* (**15**). Following route B, and starting from **45** (0.19 g, 1.0 mmol) 4-(dimethylamino)benzaldehyde (0.16 g, 1.1 mmol) a crude of reaction was obtained that was purified by column chromatography (PE/EtOAc 7:3), and crystallized from EtOH to obtain **15** as a red solid (0.20 g), 62% yield, mp 137–149 °C. ^1^H-NMR δ 2.57 (t, *J* = 2.4 Hz, 1H, CH), 3.04 (s, 6H, CH_3_), 3.89 (s, 3H, OCH_3_), 4.76 (d, *J* = 2.4 Hz, 2H, OCH_2_), 6.60 (s, 1H, H-3), 6.63 (dd, *J*= 2.4 and 10.4 Hz, 1H, H-5), 6.69 (d, *J* = 8.8 Hz, 2H, H-6′ and H-2′), 7.26 (d, *J* = 15.6 Hz, 1H, β-CH=), 7.63 (d, *J* = 15.6 Hz, 1H, α-CH=), 7.69 (d, *J* = 8.8 Hz, 1H, H-6). ^13^C-NMR δ 41.34, 56.18, 55.21, 76.44, 78.76, 101.38, 106,96, 111.15, 113.24, 118.91, 123.96, 124.99, 145.12, 152.45, 163.07, 166.21, 198.10. ESI-MS (*m/z*) 336 (M + 1). HRMS ESI + [M + 1]: calcd for C_21_H_21_NO_3_, 336.1599. Found: 336.1597.

*(E)-3-(4-(dimethylamino)phenyl)-1-(2-hydroxy-4-(prop-2-yn-1-yloxy)phenyl)prop-2-en-1-one* (**17**). Following route B, and starting from **42** (0.20 g, 1.0 mmol) 4-(dimethylamino)benzaldehyde (0.16 g, 1.1 mmol), a crude of reaction was obtained that was purified by column chromatography (PE/EtOAc 7:3), and crystallized from EtOH to obtain **17** as a red solid (0.20 g), 62% yield, mp 137–149 °C. ^1^H-NMR δ 2.59 (t, *J* = 2.4 Hz, 1H, CH), 3.92 (s, 3H, OCH_3_), 4.78 (s, 2H, OCH_2_), 6.61 (d, *J* = 2.0 Hz, 1H, H-3), 6.66 (dd, *J* = 2.4 and 8.8 Hz, 1H, H-5), 7.28 (dd, *J* = 2.2 and 8.4 Hz 1H, H-5′), 7.45 (d, *J* = 2.2 Hz, 1H, H-3′), 7.47 (d, *J* = 16.0 Hz, 1H, β-CH=), 7.64 (d, *J* = 8.8 Hz, 1H, H-6′), 7.79 (d, *J* = 8.8 Hz, 1H, H-6), 7.98 (d, *J* = 16.0 Hz, 1H, α-CH=). ^13^C-NMR δ 55.81, 56.71, 76.42, 77.66, 101.10, 107.54, 121.56, 122.47, 124.94, 125.98, 127.15, 129.74, 130.45, 1.3.31, 136.60, 161.74, 167.21, 194.11. ESI-MS (*m/z*) 322 (M + 1). HRMS ESI + [M + 1]: calcd for C_20_H_19_NO_3_, 322.1443. Found: 322.1445.

*(E)-3-(2,4-dichlorophenyl)-1-(2-hydroxy-4-((3-methylbut-2-en-1-yl)oxy)phenyl)prop-2-en-1-one* (**19**). Following route A, and starting from **41** (0.22 g, 1.0 mmol) and 2,4-dichlorobenzaldehyde (0.19 g, 1.1 mmol) a solid product was obtained that was collected by vacuum filtration and crystallized from EtOH to give **19** as a pale yellow solid (0.33 g), 88% yield, mp 191–93 °C. ^1^H-NMR δ 1.77 (s, 3H, CH_3_), 1.83 (s, 3H, CH_3_), 4.59 (d, *J* = 6.8 Hz, 2H, OCH_2_), 5.50 (t, *J* = 6.8 Hz, 1H, CH), 6.51 (d, *J* = 7.2 Hz, 1H, H-5), 6.52 (s, 1H, H-3), 7.32 (dd, *J* = 2.2 and 6.8 Hz, 1H, H-5′), 7.50 (d, *J* = 2.0 Hz, 1H, H-3′), 7.69 (d, *J* = 8.8 Hz, 1H, H-6′), 7.55 (d, *J* = 15.6 Hz, 1H, β-CH=), 7.80 (d, *J* = 9.2 Hz, 1H, H-6), 8.19 (d, *J* = 15.6 Hz, 1H, α-CH=), 13.3 (s, 1H, OH). ^13^C-NMR δ 18.23, 24.67, 64.64, 103.12, 107.75, 113.93, 119.45, 121.43, 124.11, 128.31, 130.00, 131.05, 131. 77, 138.54, 145.46, 165.92, 169.03, 194.34. ESI-MS (*m/z*) 377 (M + 1). HRMS ESI + [M + 1]: calcd for C_20_H_18_Cl_2_O_3_, 377.0711. Found: 377.0713.

*(E)-1-(2,4-bis(prop-2-yn-1-yloxy)phenyl)-3-(2,4-dichlorophenyl)prop-2-en-1-one* (**23**). Following route A, and starting from **47** (0.23 g, 1.0 mmol) and 2,4-dichlorobenzaldehyde (0.19 g, 1.1 mmol) a solid product was obtained that was collected by vacuum filtration and crystallized from EtOH to give **23** as a pale yellow solid (0.30 g), 78% yield, mp 166–68 °C. ^1^H-NMR δ 2.58-2.62 (m, 2H, CH), 4.77 (d, *J* = 2.4 Hz, 2H, OCH_2_), 4.79 (d, *J* = 2.4 Hz, 2H, OCH_2_), 6.70 (d, *J* = 2.0 Hz, 1H, H-3), 6.73 (dd, *J* = 2.0 and 8.0 Hz, 1H, H-5), 7.26 (dd, *J* = 2.0 and 8.0 Hz, 1H, H-5′), 7.45 (d, *J* = 2.0 Hz, 1H, H-3′), 7.54 (d, *J* = 16.0 Hz, 1H, β-CH=), 7.70 (d, *J* = 8.4 Hz, 1H, H-6′), 7.82 (d, *J* = 8.4 Hz, 1H, H-6), 7.99 (d, *J* = 15.6 Hz, 1H, α-CH=). ^13^C-NMR δ 56.44, 75.16, 78.92, 101.33, 107.21, 130.55, 121.98, 124.27, 126.19, 127.54, 128.23, 131.29, 137.37, 148.32, 162.62, 166.88, 193.71. ESI-MS (*m/z*) 385 (M + 1). HRMS ESI + [M + 1]: calcd for C_21_H_14_Cl_2_O_3_, 385.0398. Found: 385.0398.

*(E)-1-(2,4-bis(prop-2-yn-1-yloxy)phenyl)-3-(4-(dimethylamino)phenyl)prop-2-en-1-one* (**27**). Following route B, and starting from **47** (0.23 g, 1.0 mmol) and 4-(dimethylamino)benzaldehyde (0.16 g, 1.1 mmol), a crude of reaction was obtained that was purified by column chromatography (PE/EtOAc 7:3), and crystallized from EtOH to obtain to obtain **27** as a red solid (0.19 g), 55% yield, mp 127–129 °C. ^1^H-NMR δ 2.56–2.61 (m, 2H, CH), 3.02 (s, 3H, CH_3_), 3.02 (s, 3H, CH_3_), 4.72–4.78 (m, 4H, OCH_2_), 6.53 (dd, *J* = 2.0 and 8.4 Hz, 1H, H-5), 6.56 (d, *J* = 2.4 Hz, 1H, H-3), 6.7(d, *J* = 8.4 Hz, 2H, H-3′ and H-5′), 7.36 (d, *J* = 15.2 Hz, 1H, β-CH=), 7.58 (d, *J* = 8.4 Hz, 2H, H-2′ and H-6′), 7.87 (d, *J* = 8.8 Hz, 1H, H-6), 7.89 (d, *J* = 15.2 Hz, 1H, α-CH=). ^13^C-NMR δ 41.41, 41.49, 56.43, 56.63, 76.11, 76.32, 78.78, 78.92, 101.33, 107.64, 111.32, 118.32, 118.54, 123.564, 124.32, 129.95, 131.12, 145.63, 151.01, 162.90, 165.17, 196.15. ESI-MS (*m/z*) 360 (M + 1). HRMS ESI + [M + 1]: calcd for C_23_H_21_NO_3_, 360.1599. Found: 360.1597.

*(E)-3-(4-Fluorophenyl)-1-(2-methoxy-4-(2-(4-phenylpiperazin-1-yl)ethoxy)phenyl)prop-2-en-1-one* (**28**). Following route A, and starting from **48** (0.35 g, 1.0 mmol) and 4-fluorobenzaldehyde (0.14 g, 1.1 mmol), a semisolid product was obtained that was extracted with DCM (3 × 30 mL). The unified organic layers were dried over anhydrous Na_2_SO_4_ and evaporated to dryness to give a crude that was purified by column chromatography (DCM/MeOH 95:5), to produce **28** as a semisolid product (0.15 g), 32% yield, that was converted into the hydrochloride salt, mp 174–176 °C. ^1^H-NMR δ 3.41–3.45 (m, 2H, piperazine), 3.61–3.45 (m, 2H, piperazine), 3.91–3.95 (m, 4H, piperazine), 3.96 (s, 3H, OCH_3_), 4.19 (t, *J* = 5.6 Hz, 2H, OCH_2_), 4.89(t, *J* = 5.6 Hz, 2H, NCH_2_), 6.55 (dd, *J* = 8.4 and 2.2 Hz, 1H, H-5), 6.62 (d, *J* = 2.2 Hz, 1H, H-3), 6.94–7.06 (m, 1H, Ph), 7.06–7.11 (m, 2H, Ph), 7.34–7.38 (m, 2H, Ph), 7.36 (d, *J* = 15.6 Hz, 1H, β-CH=), 7.52–7.56 (m, 2H, H-2′ and H-6′), 7.61 (d, *J* = 15.6 Hz, 1H, α-CH=), 7.69 (d, *J* = 8.8 Hz, 1H, H-6). ^13^C-NMR δ 49.27, 53.81, 57.03, 58.47, 66.49, 101.67, 108.12, 114.19, 116.25, 116.47, 119.94, 128.75, 129.26, 130.80, 136.24, 132.45, 145.16, 151.40, 165.36. ESI-MS (*m/z*) 461 (M + 1). HRMS ESI + [M + 1]: calcd for C_28_H_29_FN_2_O_3_, 461.2240. Found: 461.2142.

*(E)-3-(4-Fluorophenyl)-1-(4-hydroxy-2-methoxyphenyl)prop-2-en-1-one* (**29**). Following route A, and starting from 1-(4-hydroxy-2-methoxyphenyl)ethan-1-one (0.17 g, 1.0 mmol) and 4-fluorobenzaldehyde (0.14 g, 1.1 mmol), a semisolid product was obtained that was extracted with EtOAc (3 × 30 mL). The unified organic layers were dried over anhydrous Na_2_SO_4_ and evaporated to dryness to give a crude that was purified by column chromatography (PE/EtOAc 9:1), and crystallized from EtOH to produce **29** as a solid (0.035 g), 13% yield, mp 166–168 °C. ^1^H-NMR δ 3.89 (s, 3H, OCH_3_). 6.50 (dd, *J* = 2.2 and 8.4 Hz, 1H, H-5), 6.51 (d, *J* =2.2 Hz, 1H, H-3), 7.06-7.10 (m, 2H, H-3′and H-5′), 7.44 (d, *J* = 16.0 Hz, 1H, β-CH=), 7.55-7.58 (m, 2H, H-2′ and H-6′), 7.61 (d, *J* = 16.0 Hz, 1H, β-CH=), 764 (d, *J* = 15.6 Hz, 1H, α-CH=), 7.69 (d, *J* = 8.8 Hz, 1H, H-6). ^13^C-NMR δ 102.64, 113.45, 115.24, 115.28, 118.67, 130.25, 130.56, 145.21, 162.49, 162.77, 165.45, 168.88. ESI-MS (*m/z*) 273 (M + 1). HRMS ESI + [M + 1]: calcd for C_16_H_13_FO_3_, 273.0927. Found: 273.0925.

*(E)-3-(4-fluorophenyl)-1-(4-((1-(2-hydroxyethyl)-1H-1,2,3-triazol-4-yl)methoxy)-2-methoxyphenyl)prop-2-en-1-one* (**30**). Following route A, and starting from **49** (0.29 g, 1.0 mmol) and 4-fluorobenzaldehyde (0.14 g, 1.1 mmol), a solid product was obtained that was collected by vacuum filtration and purified by column chromatography (PE/EtOAc 1:1), to produce **30** as a white solid (0.079 g), 20% yield, mp 100–102 °C. ^1^H-NMR δ 2.93 (br, 1H, OH), 3.88 (s, 3H, OCH_3_), 4.10 (t, *J* = 5.2 Hz, 2H, NCH_2_), 4.55 (t, *J* = 5.2 Hz, 2H, CH_2_OH), 5.21 (s, 2H, OCH_2_), 6.52 (d, *J* = 2.4 Hz, 1H, H-3), 6.62 (dd, *J* = 2.4 and 7.6 Hz, 1H, H-5), 7.42–7.49 (m, 2H, H-3′and H-5′), 7.60 (d, *J* = 15.6 Hz, 1H, β-CH=), 7.63–7.69 (m, 2H, H-2′ and H-6′), 7.80 (s, 1H, triazole CH), 7.74 (d, *J* = 15.6 Hz, 1H, α-CH=), (7.83 (d, *J* = 8.0 Hz, 1H, H-6). ^13^C-NMR δ 54.11, 55.64, 60.57, 72.12, 100.54, 105.21, 115.64, 115.74, 118.52, 123.77, 127.32, 130.47, 131.25, 131.55, 142.84, 145.37, 145.22, 162.34, 167.88, 195.94. ESI-MS (*m/z*) 398 (M + 1). HRMS ESI + [M + 1]: calcd for C_21_H_20_FN_3_O_4_, 398.1516. Found: 398.1515.

*(E)-3-(4-fluorophenyl)-1-(4-(2-hydroxyethoxy)phenyl)prop-2-en-1-one* (**31**). Following route A, and starting from **50** (0.18 g, 1.0 mmol) and 4-fluorobenzaldehyde (0.14 g, 1.1 mmol), a solid product was obtained that was collected by vacuum filtration and purified by column chromatography (PE/EtOAc 4:1), to produce **31** as a white solid (0.19 g), 67% yield. ^1^H-NMR (acetone d_6_) δ 3.92–3.96 (m, 2H, CH_2_OH), 4.02–4.04 (m, 1H, OH), 4.18 (t, *J* = 6.4 Hz, 2H, CH_2_O), 7.05 (d, *J* = 6.8 Hz, 2H, H-3 and H-5), 7.20 (t, *J* = 8.8 Hz, 2H, H-3′ and H-5′), 7.72 (d, *J* = 15.6 Hz, 1H, β-CH=), 7.81 (d, *J* = 15.6 Hz, 1H, α-CH=), 7.83-7.88 (m, 2H, H-2′ and H-6′), 8.12 (d, *J* = 6.8 Hz, 2H, H-2 and H-6). ^13^C-NMR δ (acetone d_6_) 60.9, 69.9, 114.12, 116.80, 120.54, 129.65, 130.22, 130.97, 131.15, 131.45, 145.16, 160.21, 168. 01. ESI-MS (*m/z*) 287 (M + 1). HRMS ESI + [M + 1]: calcd for C_17_H_15_FO_3_, 287.1083. Found: 287.1085.

*(E)-1-(4-((3-methylbut-2-en-1-yl)oxy)phenyl)-3-(pyridin-3-yl)prop-2-en-1-one* (**32**). Following Route C, and starting from **51** (0.20 g, 1.0 mmol) and pyridine-3-carboxyaldehyde (0.12 g, 1.1 mmol) a solid product was obtained that was collected by vacuum filtration and crystallized from EtOH to give **32** (0.17 g), 60% yield, mp 87–88 °C. ^1^H-NMR δ 1.78 (s, 3H, CH_3_), 1.83 (s, 3H, CH_3_), 4.62 (d, *J* = 6.8 Hz, 2H, OCH_2_), 5.11 (t, *J* = 1.2 Hz, 1H, CH), 7.01 (d, *J* = 8.8 Hz, 2H, H-3 and H-5), 7.35-7.39 (m, 1H, H-5′), 7.62 (d, *J* = 15.6 Hz, 1H, β-CH=), 7.79 (d, *J* = 15.6 Hz, 1H, α-CH=), 7.95 (dd, *J* = 1.6 and 7.6 Hz, 1H, H-4′), 8.05 (d, *J* = 8.8 Hz, 2H, H-2 and H-6), 8.63 (dd, *J* = 1.6 and 7.6 Hz, 1H, H-4′), 8.87 (d, *J* = 2.0 Hz, 1H, H-2′). ^13^C-NMR δ 54.21, 55.87, 60.02, 72.15, 101.33, 105.74, 113.11, 118.21, 123.44, 128.64, 130.21, 130.31, 130.56, 131.64, 142.85, 162.18, 162.47. 163.54, 167.04, 195.32. ESI-MS (*m/z*) 294 (M + 1). HRMS ESI + [M + 1]: calcd for C_19_H_19_NO_2_, 294.1494. Found: 294.1495.

*(E)-1-(4-((3-methylbut-2-en-1-yl)oxy)phenyl)-3-(pyridin-4-yl)prop-2-en-1-one* (**33**). Following Route C, and starting from **51** (0.20 g, 1.0 mmol) and pyridine-4-carboxyaldehyde (0.12 g, 1.1 mmol) a solid was obtained that was filtered under vacuum and crystallized from EtOH to give **33** (0.21 g), 77% yield, mp 91–93 °C. ^1^H-NMR δ 1.78 (s, 3H, CH_3_), 1.83 (s, 3H, CH_3_), 4.62 (d, *J* = 7.2 Hz, 2H, OCH_2_), 5.51 (t, *J* = 6.8 Hz, 1H, CH), 7.01 (d, *J* = 8.8 Hz, 2H, H-3 and H-5), 7.48 (d, *J* = 7.2 Hz, 2H, H-2′ and H-6′), 7.63 (d, *J* = 15.6 Hz, 1H, β-CH=), 7.71 (d, *J* = 15.6 Hz, 1H, α-CH=), 8.04 (d, *J* = 8.4 Hz, 2H, H-2 and H-6), 8.69 (d, *J* = 7.2 Hz, 2H, H-3′ and H-5′). ^13^C-NMR δ 18.32, 18.57, 21.41, 64.21, 114.22, 119.65, 123.74, 127.88, 130.51, 138.08, 144.00, 149.16, 164.77, 191.66. ESI-MS (*m/z*) 294 (M + 1). HRMS ESI + [M + 1]: calcd for C_19_H_19_NO_2_, 294.1494. Found: 294.1492.

*(E)-1-(4-((3-methylbut-2-en-1-yl)oxy)phenyl)-3-(4-nitrophenyl)prop-2-en-1-one* (**34**). Following Route C and starting from **51** (0.20 g, 1.0 mmol) and of 4-nitrobenzaldheyde (0.17 g; 1.1 mmol) a solid was obtained that was filtered under vacuum and purified by crystallization from EtOH to give **34** as a brown powder, (0.26 g) 70% yield, mp 100–102 °C. ^1^H-NMR δ 1.79 (s, 3H, CH_3_), 1.85 (s, 3H, CH_3_), 4.62 (d, *J* = 7.2 Hz, 2H, OCH_2_), 5.51 (t, *J* = 6.8 Hz, 1H, CH), 7.00 (d, *J* = 8.4 Hz, 2H, H-3 and H-5), 7.63 (d, *J* = 15.6 Hz, 1H, β-CH=), 7.79 (d, *J* = 8.0 Hz, 2H, H-2′ and H-6′), (d, *J* = 15.6 Hz, 1H, β-CH=), 7.90 (d, *J* = 15.6 Hz, 1H, α-CH=), 8.07 (d, *J* = 9.2 Hz, 2H, H-2 and H-6), 8.29 (d, *J* = 8.8 Hz, 2H, H-3′ and H-5′). ^13^C-NMR δ 18.52, 24.11, 64.08, 114.63, 119.08, 121.08, 123.87, 129.45, 129.95, 138.11, 145.36, 163.21, 189.32. ESI-MS (*m/z*) 338 (M + 1). HRMS ESI + [M + 1]: calcd for C_20_H_19_NO_4_, 338.1392. Found: 338.1394.

*(E)-3-(4-(dimethylamino)phenyl)-1-(4-((3-methylbut-2-en-1-yl)oxy)phenyl)prop-2-en-1-one* (**35**). Following Route B, and starting from **51** (0.20 g, 1.0 mmol) and 4-(dimethylamino)benzaldehyde (0.16 g, 1.1 mmol), a red solid was obtained that was filtered under *vacuum* and crystallized from EtOH to give **35** as a red powder (0.03 g), 15% yield, 121–123 °C. ^1^H-NMR δ 1.77 (s, 3H, CH_3_), 1.82 (s, 3H, CH_3_), 3.05 (s, 6H, NCH_3_), 4.60 (d, *J* = 6.4 Hz, 2H, OCH_2_), 5.51 (t, *J* = 6.8 Hz, 1H, CH), 6.70 (d, *J* = 8.4 Hz, 2H, H-2′ and H-6′), 6.98 (d, *J* = 8.8 Hz, 2H, H-3 and H-5), 7.36 (d, *J* = 15.6 Hz, 1H, β-CH=), 7.55 (d, *J* = 9.2 Hz, 2H, H-3′ and H-5′), 7.79 (d, *J* = 15.6 Hz, 1H, α-CH=), 8.02 (d, *J* = 8.8 Hz, 2H, H-2 and H-6). ^13^C-NMR δ 18.8, 24.36, 41.35, 64.02, 11.33, 111.45, 111.66, 114.25, 119.57, 121.18, 124.61, 129.72, 130.12, 130.97, 131.25, 145.74, 150.01, 163.75, 189.22. ESI-MS (*m/z*) 336 (M + 1). HRMS ESI + [M + 1]: calcd for C_22_H_25_NO_2_, 336.1963. Found: 336.1964.

*(E)-1-(4-(prop-2-yn-1-yloxy)phenyl)-3-(pyridin-3-yl)prop-2-en-1-one* (**36**). Following Route C, and starting from **52** (0.17 g, 1.0 mmol) and pyridine-3-carboxyaldehyde (0.12 g, 1.1 mmol) a solid was obtained that was filtered under vacuum and crystallized from EtOH to give **36** (0.17 g), 67% yield, mp 96–98 °C. ^1^H-NMR δ 2.58 (d, *J* = 2.0 Hz, 1H, CH), 4.82 (d, *J* = 2.4 Hz, 2H, OCH_2_), 7.09 (d, *J* = 9.2 Hz, 2H, H-3 and H-5), 7.33–7.39 (m, 1H, H-5′), 7.61 (d, *J* = 16.0 Hz, 1H, β-CH=), 7.80 (d, *J* = 15.6 Hz, 1H, α-CH=), 7.93 (dd, *J* = 1.6 and 8.8 Hz, 1H, H-4′), 8.07 (d, *J* = 8.8 Hz, 2H, H-2 and H-6), 8.64 (dd, *J* = 1.6 and 8.8 Hz, 1H, H-3′), 8.87 (d, *J* = 1.6 Hz, 1H, H-2′). ^13^C-NMR δ 56.98, 76.11, 87.91, 114.22, 114.65, 123.45. 123.87, 129.64, 130.45, 130.76, 132.16, 141.74, 142.03, 149.84, 163.25, 189.99. ESI-MS (*m/z*) 264 (M + 1). HRMS ESI + [M + 1]: calcd for C_17_H_13_NO_2_, 264.1024. Found: 264.1022.

*(E)-1-(4-(prop-2-yn-1-yloxy)phenyl)-3-(pyridin-4-yl)prop-2-en-1-one* (**37**). Following Route C, and starting from **52** (0.17 g, 1.0 mmol) and pyridine-4-carboxyaldehyde (0.12 g, 1.1 mmol) a solid was obtained that was filtered under vacuum and crystallized from EtOH to give **37** (0.20 g), 80% yield, mp 99–101 °C. ^1^H-NMR δ 2.57 (t, *J* = 2.0 Hz, 1H, CH), 4.79 (d, *J* = 2.4 Hz, 2H, OCH_2_), 7.09 (d, *J* = 9.2 Hz, 2H, H-3 and H-5), 7.47 (d, *J* = 8.0 Hz, 2H, H-2′ and H-6′), 7.66 (d, *J* = 16.4 Hz, 1H, β-CH=), 7.70 (d, *J* = 16.4 Hz, 1H, α-CH=), 8.06 (d, *J* = 8.8 Hz, 2H, H-2 and H-6), 8.69 (d, *J* = 8.6 Hz, 2H, H-3′ and H-5′). ^13^C-NMR δ 56.41, 76.11, 78.78, 114.21, 114.68, 123.54, 123.98, 124.54, 127.88, 128.45, 130.02. 130.64, 131.11, 144.56, 149.00, 149.857, 163.24, 189.69. ESI-MS (*m/z*) 264 (M + 1). HRMS ESI + [M + 1]: calcd for C_17_H_13_NO_2_, 264.1024. Found: 264.1025.

*(E)-3-(4-nitrophenyl)-1-(4-(prop-2-yn-1-yloxy)phenyl)prop-2-en-1-one* (**38**). Following Route C, and starting from **52** (0.17 g, 1.0 mmol) and 4-nitrobenzaldheyde (0.17 g; 1.1 mmol) a solid was obtained that was filtered under vacuum and crystallized from EtOH to give **38** as a brown powder, (0.16 g) 54% yield, mp 103–105 °C. ^1^H-NMR δ 2.58 (d, *J* = 2.0 Hz, 1H, CH), 4.82 (d, *J* = 2.4 Hz, 2H, OCH_2_), 7.09 (d, *J* = 8.4 Hz, 2H, H-3 and H-5), 7.64 (d, *J* = 15.6 Hz, 1H, β-CH=), 7.78 (d, *J* = 8.8 Hz, 2H, H-2′ and H-6′), 7.80 (d, *J* = 15.6 Hz, 1H, α-CH=), 8.07 (d, 2H, H-3′ and H-5′), 8.87 (s, 1H, H-2), 8.07 (d, *J* = 9.2 Hz, 2H, H-2 and H-6), 8.28 (d, *J* = 8.8 Hz, 2H, H-3′ and H-5′). ^13^C-NMR (CDCl_3_) δ 56.21, 76.54, 78.11, 114.77, 121.18, 123.87, 129.11, 130.54, 141.07, 145.98, 147.64, 163.74, 189.60. ESI-MS (*m/z*) 308 (M + 1). HRMS ESI + [M + 1]: calcd for C_18_H_13_NO_4_, 308.0923. Found: 308.0922.

*(E)-3-(4-(dimethylamino)phenyl)-1-(4-(prop-2-yn-1-yloxy)phenyl)prop-2-en-1-one* (**39**). Following Route B, and starting from **52** (0.17 g, 1.0 mmol) and 4-(dimethylamino)benzaldehyde (0.16 g, 1.1 mmol), a red solid was obtained that was filtered under vacuum and crystallized from EtOH to give **39** as a red powder (0.03 g), 15% yield, 133–135 °C. ^1^H-NMR δ 2.56 (t, *J* = 2.4 Hz, 1H, CH), 3.05 (d, 6H, NCH_3_), 4.82 (d, *J* = 2.8 Hz, 2H, OCH_2_), 6.70 (d, *J* = 8.4 Hz, 2H, H-3 and H-5), 7.05 (d, *J* = 9.2 Hz, 2H, H-3′ and H-5′), 7.35 (d, *J* = 15.6 Hz, 1H, β-CH=), 7.55 (d, *J* = 8.8 Hz, 2H, H-2′ and H-6′), 7.80 (d, *J* = 15.6 Hz, 1H, α-CH=), 8.04 (d, *J* = 8.8 Hz, 2H, H-2 and H-6). ^13^C-NMR δ 41.12,56.21, 76.44, 78.64, 111.10, 114.26, 121.87, 124.68, 129.39, 129.85, 130.22, 130.51, 145.74, 150.63, 164.08, 189.89. ESI-MS (*m/z*) 306 (M + 1). HRMS ESI + [M + 1]: calcd for C_20_H_19_NO_2_, 306.1494. Found: 306.1492.

*(Z)-6-(prop-2-yn-1-yloxy)-2-(pyridin-3-ylmethylene)benzofuran-3(2H)-one* (**40**). To a solution of **16** (0.17 g, 0.60 mmol) in EtOH (17 mL) at 0 °C, NaOH aq solution (0.16 g, 3.33 mmol, dissolved in 1 mL of H_2_O) and H_2_O_2_ (0.07 mL, 3.33 mmol) were added dropwise. The reaction was stirred at rt for 12 h, then water was added and the solution was acidified with 2N HCl. The solid formed was separated by vacuum filtration, dried, and purified by column chromatography (EtOAc/MeOH 9:1) to obtain **40** as a red solid (0.05 g), 29% yield, mp 121.123 °C. ^1^H-NMR δ 3.42 (t, *J* = 2.4, 1H, CH), 4.69 (d, *J* = 2.4 Hz, 2H, OCH_2_), 6.26 (d, *J* = 8.8 Hz, 1H, H-5), 6.28 (s, 1H, H-7), 7.31-7.34 (m, 1H, H-5′), 7.59 (d, *J* = 8.4 Hz, 1H, H-4), 8.12 (d, *J* = 8.0 Hz, 1H, H-6′), 8.38 (s, 1H, CH), 8.46 (s, 1H, H-4′), 8.91 (S, 1H, H-2′). ^13^C-NMR δ 56.21, 76.11, 78.84, 100.00, 109.54, 112.57, 114.08, 123.22, 125.08, 132.11, 132.55, 132.98, 148.64, 149.74, 152.32, 165.09, 167.46, 18.02. ESI-MS (*m/z*) 278 (M + 1)). HRMS ESI + [M + 1]: calcd for C_17_H_11_NO_3_, 278.0817. Found: 278.0815.

### 4.2. Yeast Species and Identification

The yeasts included in the present study were *Candida albicans* ATCC 10231 (American Type Culture Collection) and a collection of yeasts recovered from a variety of clinical specimens, i.e., blood, urine, genital swabs, bronchoalveolar lavage, nail specimens, collected at the Microbiology Unit, St. Orsola Malpighi University Hospital, Bologna, Italy. The clinical strain isolates were identified by standard procedures, including colony morphology on chromogenic agar (CHROMagar *Candida* medium, Becton Dickinson, Heidelberg, Germany) and confirmed by MALDI Biotyper System using matrix-assisted laser desorption ionization–time of flight mass spectrometry (MALDI-TOF MS, Bruker Daltonik, GmbH, Germany) [28]. The distribution of isolates is reported in Table 1 and Table 2.

### 4.3. In Vitro Susceptibility Testing

The in vitro antifungal activity of the chalcones was evaluated by a microdilution broth method in accordance with the guidelines provided by the EUCAST Edef 7.3 [22]. Briefly, all strains were cultured on Sabauraud dextrose, then inocula, prepared at 0.5 McFarland, were diluted 1:20 in RPMI-1640 medium (Gibco^®^, ThermoFisher Scientific Inc., Waltham, MA, USA), containing glucose 2%, 0.3% levo-glutamine buffered to pH 7.0 with 0.165 M 3-(N-morpholino)propanesulfonic acid (MOPS) to yield 1-5 × 10^5^ cells/mL. A total of 100 µL of the yeast suspension was inoculated in a 96-well microplate and incubated with 100 µL of serially 2-fold dilution of the compound in the range 200–6.25 µM. The following controls were included: yeast suspensions incubated in RPMI-1640 culture medium (positive growth control), or supplemented with dilutions of DMSO, or fluconazole (Sigma-Aldrich, St. Louis, MO, USA) as a commercial drug. To check the background turbidity of reagents and the sterility of the procedure, dilutions of each compound were incubated in RPMI-1640 culture medium, without yeast inoculum. The inoculated plate was incubated at 37 °C for 24 h, and subsequently the optical density at 630 nm was measured by the Multiskan Ascent microplate reader (Thermo Fisher Scientific Inc.). The effectiveness of the compounds was expressed as percent inhibition relative to the positive growth control when the inhibition yielded the 50% at 100 µM. The IC_50_ value was determined by interpolation of the dose–response curves generated by plotting the percentages of growth inhibition, relative to the drug-free control (set to 100% of growth), as a function of the tested concentrations (on a logarithm scale). Statistical analysis was carried out by the nonlinear regression method using GraphPad Prism version 5.0 for Windows (GraphPad Software, San Diego, CA, USA). All assays were performed in triplicate and at least two independent experiments were carried out.

### 4.4. Effect on Yeast-to-Hyphae Transition

A suspension of *C. albicans* ATCC 10231 (5 × 10^5^ cells/mL) was prepared in RPMI-1640 culture medium supplemented with 10% (*v*/*v*) FCS (fetal calf serum) and incubated for 2 h at 37 °C with chalcones **1**, **3**–**5**, **7**, **14**, and **28** at IC_50_ values, in a 48-well flat-bottom polystyrene microplate. Blastospore and hyphae forms were observed under a light microscope. Cells from at least two different fields of view were observed and were considered to be germinated if they had a germ tube at least twice the length of the cell diameter [29].

### 4.5. Agar-Invasive Hyphal Growth

For the “*Spider*” agar assay, a medium consisted in nutrient broth 1% (*w*/*v*), D-mannitol 1% (*w*/*v*), K_2_HPO_4_ 0.2% (*w*/*v*) and agar 1.35% (*w*/*v*) with the addition of chalcones **1**, **3**–**5**, **7**, **14**, and **28** at IC_50_ values was used [29]. Aliquots (2 µL) of a suspension of *C. albicans* ATCC 10231 (5 × 10^5^ cells/mL) were spotted onto medium in triplicate and the morphology of growing colonies was observed following three days of incubation at 37 °C. Spider agar plates without chalcones, containing DMSO, served as the control. The presence of filaments at the colony edges was determined using a light microscope under 10× magnification.

### 4.6. Biofilm Investigation

For biofilm formation, a suspension of *C. albicans* ATCC 10231 at 5 × 10^5^ cells/mL was prepared in RPMI-1640 culture medium and aliquots of 100 µL were transferred to a 96-well flat-bottom polystyrene microplate. Cell cultures were treated with 100 µL of chalcones **1**, **3**–**5**, **7**, **14**, and **28** at IC_50_ values, and incubated statically for 24 h at 37 °C to allow biofilm formation. Following the attachment phase, the free-floating cells were removed, and biofilms were carefully washed with 200 µL of PBS to remove planktonic fungi. The biofilm mass was quantified by crystal violet (CV) staining. Briefly, 200 µL of CV solution (0.1% in water) were added to each well containing a completely air-dried biofilm, and incubated for 30 min at 37 °C. Then wells were washed twice with water to remove the unbound dye and CV was dissolved with 200 µL of 95% ethanol for 30 min [30]. Finally, 150 µL of the colored supernatant from each well were transferred to a new microplate and the absorbance was read at 550 nm. The percentage inhibition of biofilm formation for each well containing the compound was calculated by comparing the biofilm mass formed in the absence of test agent. Six wells for each treatment regimen were prepared and the experiment was performed twice. Data were reported as mean OD values ± standard deviation (SD) and one-way analysis of variance (ANOVA), followed by Dunnett’s multiple comparison test to compare data among the different experimental conditions; statistically significant differences were determined at *p* < 0.05.

### 4.7. Cytotoxicity Tests

African green monkey kidney cells (Vero ATCC CCL-81) were cultured in Eagle’s Minimal Essential Medium (MEM) (Sigma-Aldrich), supplemented with 10% fetal bovine serum (FBS) (Carlo Erba Reagents, Milan, Italy), 100 U/mL penicillin, and 100 µg/mL streptomycin at 37 °C with 5% CO_2_. For experiments, cells were seeded into 96-well plates at 10^4^ cells/well, and incubated at 37 °C for 24 h. Following washes with PBS, cell monolayer was incubated with 100 µL of serially 2-fold dilution of the compound in the range 200–6.25 µM. Both untreated cells and cells incubated with medium containing solvent dilutions were included in each experiment as controls.

The cell viability was assessed by a WST8-based assay according to the manufacturer’s instructions (CCK-8, Cell Counting Kit-8, Dojindo Molecular Technologies, Rockville, MD, USA). After 72 h of incubation, culture medium was removed from each well, the monolayer was washed with PBS, and 100 µL of fresh medium containing 10 µL of CCK-8 solution were added. Following a 2 h at 37 °C, the absorbance was measured at 450/630 nm; data were calculated as the percentage of the cell viability relative to the untreated controls. 

## 5. Conclusions

In the present study a small library of 40 chalcone-based analogues, differently functionalized on both A-and B-rings, were evaluated in vitro for their antifungal potential. Interestingly, sixteen chalcones showed a good inhibitory activity against *C. albicans* control strain associated with a different degree of cytotoxicity towards mammalian cells. Among them, a subset of seven derivatives (**1**, **3**–**5**, **7**, **14**, and **28**) exerted a low systemic toxicity, underlying a favorable SI, i.e., degree of host/fungi selectivity. These selective inhibitors were then further investigated regarding their spectrum of activity (collection of *Candida* spp. and non-*Candida* spp. isolates obtained from different clinical specimens) and their capability to affect microbial virulence (yeast-to-hyphae transition, filament invasion, and biofilm formation). In particular, derivative **28** demonstrated a robust dose‒response inhibitory effect against all the selected *Candida* spp. and the clinically relevant non-*Candida* yeasts, thus emerging as the most relevant antifungal agent of the series. 

On the other hand, compounds **5** and **7**, even if associated with a restricted antifungal window, showed great potential for controlling *C. albicans* pathogenesis and virulence due to their capability to interfere with the production of both hyphae and biofilm, perfectly meeting the current paradigm of anti-infective therapeutics. Indeed, these last compounds combine two modes of action by selectively interfering with growth and, as an added value, weakening microbial virulence. Indeed, disarming pathogens rather than killing them is emerging as a novel and effective strategy for infection control. 

In summary, this study allowed us to gain insight into the chemical functionalization of the chalcone involved in a favorable antifungal effect. Compounds **5**, **7**, and **28** could be regarded as valuable lead compounds worth modifying with different approaches, aimed at improving and optimizing the antimicrobial profile. 

## Figures and Tables

**Table 1:** Antifungal effect of chalcone-based analogues 1–40 against *C. albicans* control strain.

Compound	Structure	Inhibition (%) [at 100 μM]	IC_50_ [μM] ^a,b^	IC_50_ [μg/mL] ^a,b^
**1**	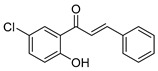	65.2 ± 3.9	52.8 [44.6–62.6]	13.7 [11.5–16.2]
**2**	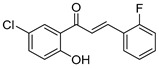	35.8 ± 1.1	n.d.	n.d.
**3**	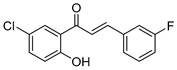	64.8 ± 1.5	57.7 [47.1–70.8]	16.0 [13.0–19.6]
**4**	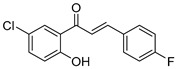	65.6 ± 3.3	48.9 [41.9–57.0]	13.5 [11.6–15.8]
**5**	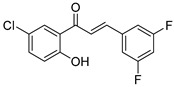	62.0 ± 1.5	51.3 [41.3–63.8]	15.1 [12.2–18.8]
**6**	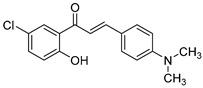	<1	n.d.	n.d.
**7**	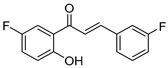	69.0 ± 12.4	53.0 [43.8–64.0]	13.8 [11.4–16.7]
**8**	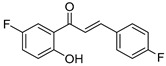	<1	n.d.	n.d.
**9**	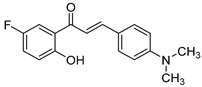	38.5 ± 2.9	n.d.	n.d.
**10 ^c^**	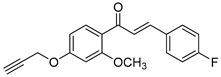	23.3 ± 9.6	n.d.	n.d.
**11**	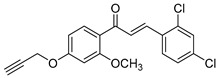	9.8 ± 6.7	n.d.	n.d.
**12 ^c^**	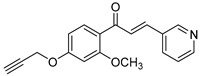	>99	30.7 [27.0–35.0]	9.0 [7.9–10.3].
**13 ^c^**	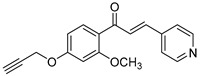	>99	8.1 [6.8–9.6]	2.4 [2.0–2.8]
**14 ^c^**	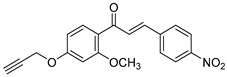	63.6 ± 6.8	39.2 [26.8–57.4]	11.5 [7.9–16.8]
**15**	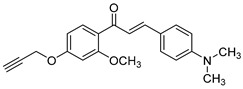	<1	n.d.	n.d.
**16 ^c^**	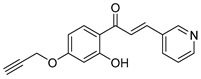	91.9 ± 8.2	10.8 [9.3–12.5]	3.2 [2.7–3.7]
**17**	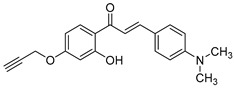	11.0 ± 3.9	n.d.	n.d.
**18 ^c^**	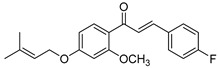	34.1 ± 7.5	n.d.	n.d.
**19**	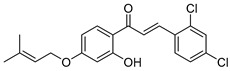	5.9 ± 5.7	n.d.	n.d.
**20 ^c^**	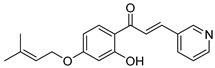	81.5 ± 4.7	16.8 [13.6–20.6]	4.9 [4.0–6.0]
**21 ^c^**	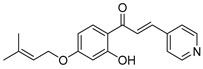	96.5 ± 1.4	12.6 [10.6–15.1]	3.7 [3.1–4.4]
**22 ^c^**	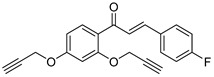	<1	n.d.	n.d.
**23**	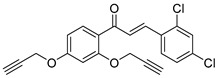	4.6 ± 13.8	n.d.	n.d.
**24 ^c^**	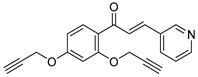	72.0 ± 9.8	43.8 [30.6–62.7]	12.8 [9.0–18.4]
**25 ^c^**	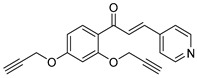	81.3 ± 17.0	26.3 [19.6–35.4]	7.7 [5.7–10.4]
**26 ^c^**	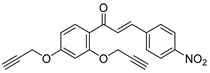	63.9 ± 3.4	36.8 [29.2–46.3]	10.8 [8.6–13.6]
**27**	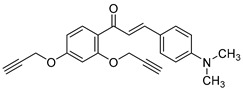	<1	n.d.	n.d.
**28**	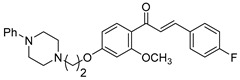	99.1 ± 0.7	55.9 [50.9–61.3]	27.7 [25.2–30.4]
**29**	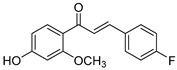	17.8 ± 4.1	n.d.	n.d.
**30**	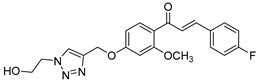	9.5 ± 4.8	n.d.	n.d.
**31**	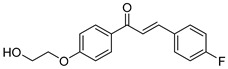	97.3 ± 0.5	12.0 [9.3–15.6]	3.5 [2.7–4.6]
**32**	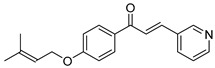	<1	n.d.	n.d.
**33**	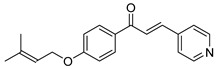	<1	n.d.	n.d.
**34**	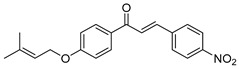	37.9 ± 5.2	n.d.	n.d.
**35**	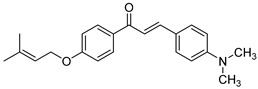	13.9 ± 3.6	n.d.	n.d.
**36**	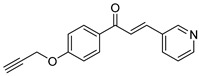	<1	n.d.	n.d.
**37**	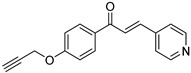	<1	n.d.	n.d.
**38**	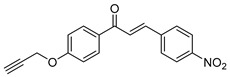	46.3 ± 3.7	n.d.	n.d.
**39**	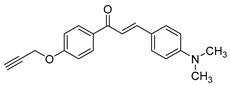	<1	n.d.	n.d.
**40**	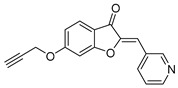	<1	n.d.	n.d.
Licochalcone ^d^			62.5–150	18.3–44.0
FLC		n.d.	0.25 [0.18–0.33]	0.07 [0.05–0.1]

^a^ IC_50_ is defined as the concentration giving rise to an inhibition of growth of 50% compared to the drug-free control. ^b^ Data are reported as mean values and 95% confidence interval. ^c^ See [17]. ^d^ See [16], FLC, fluoconazole; n.d., not determined.

**Table 2:** Cytotoxicity of selected chalcone-based analogues against Vero cells (CC_50_ values are expressed in μM and µg/mL).

Compound	CC_50_ [μM] ^a,b^	CC_50_ [μg/mL] ^a,b^
**1**	174.1 [157.0–193.1]	45 [40.6–50.0]
**3**	151.8 [131.7–175.0]	42.0 [36.4–48.4]
**4**	>200	>55.3
**5**	>200	>58.9
**7**	181.7 [126.3–261.4]	47.3 [32.9–68.0]
**12**	13.6 [12.7–14.6]	4.0 [3.7–4.3]
**13**	7.3 [6.2–8.7]	2.1 [1.8–2.5]
**14**	>200	>58.6
**16**	17.8 [15.8–20.1]	5.2 [4.6–5.9]
**20**	54.3 [50.3–58.5]	15.9 [14.8–17.2]
**21**	14.8 [13.7–16.0]	4.3 [4.0–4.7]
**24**	13.0 [11.4–14.8]	3.8 [3.3–4.3]
**25**	9.1 [8.6–9.8]	2.7 [2.5–2.9]
**26**	5.1 [4.7–5.5]	1.5 [1.4–1.6]
**28**	>200	>99.2
**31**	8.3 [7.1–9.6]	2.4 [2.1–2.8]

^a^ CC_50_ is defined as the concentration giving rise to an inhibition of growth of 50% compared to untreated Vero cells. ^b^ Data are reported as mean values and 95% confidence interval.

**Table 3:** Antifungal effect determined for compounds **1**, **3**‒**5**, **7**, **14**, **28** and for the control FLC against various *Candida* spp. The activities are expressed as IC_50_ values ^a,b^ [µM (above) and in µg/mL (below)].

Compound	*C. albicans*	*C. parapsilosis*	*C. lusitaniae*	*C. tropicalis*	*C. krusei*	*C. glabrata*	*C. utilis*
**1**	62.9 [45.9–79.8]	200	100	200	200	200	84.5 [77.5–92.2]
	16.3 [11.9–20.6]	51.7	25.9	51.7	51.7	51.7	21.9 [20.0–23.9]
**3**	100	41.5 [27.2–63.1]	38.5 [29.7–50.0]	>200	>200	>200	100
	27.7	11.5 [7.5–17.5]	10.7 [8.2–13.8]	>55.3	>55.3	>55.3	27.7
**4**	69.6 [51.7–93.7]	>200	34.6 [21.5–55.8]	200	200	200	96.7 [90.8–102.9]
	19.3 [14.3–25.9]	>55.3	9.6 [5.9–15.4]	55.3	55.3	55.3	26.8 [25.1–28.5]
**5**	100	200	47.9 [37.2–61.9]	>200	>200	>200	100
	29.5	58.9	14.1 [11.0–18.2]	>58.9	>58.9	>58.9	29.5
**7**	32.1 [26.4–39.0]	100	31.7 [26.3–38.1]	>200	100	100	100
	8.4 [6.9–10.1]	26.0	8.2 [6.8–9.9]	>52.0	26.0	26.0	26.0
**14**	31.4 [25.5–40.2]	100	48.8 [28.3–66.4]	200	200	200	33.7 [27.8–40.8]
	10.6 [21.1–19.9]	33.7	14.8 [9.6–22.4]	67.5	67.5	67.5	11.4 [9.4–13.8]
**28**	48.1 [41.2–56.0]	36.8 [30.2–44.8]	26.5 [19.9–35.2]	26.6 [24.1–29.3]	63.5 [59.1–68.3]	37.1 [31.7–43.5]	29.6 [27.0–32.4]
	23.9 [20.4–27.8]	18.3 [15.0–22.2]	13.1 [9.9–17.5]	13.2 [12.0–14.5]	31.5 [29.3–33.9]	18.4 [15.7–21.6]	14.7 [13.4–16.1]
FLC	0.42 [0.29–0.61]	0.037 [0.02–0.07]	0.05 [0.01–0.2]	0.40	64.6 [44.3–94.2]	14.4 [12.6–16.4]	1.56 [0.96–2.53]
	0.13 [0.09–0.19]	0.01 [0.01–0.02]	0.015 [0.003–0.06]	(0.31–0.52)	19.8 [13.6–28.9]	4.4 [3.9–5.0]	0.5 [0.3–0.8]

^a^ IC_50_ value is defined as the concentration giving rise to an inhibition of growth of 50% compared to the drug-free control. ^b^ Data are reported as mean values and 95% confidence interval.

**Table 4:** Antifungal effect determined for compounds **1**, **3**‒**5**, **7**, **14**, **28** and for the control FLC against various non-*Candida* yeast strains. The activities are expressed as IC_50_ values ^a,b^ [µM (above) and in µg/mL (below)].

Compound	*C. neoformans*	*S. clavata*	*R. mucilaginosa*
**1**	26.4 [22.6–30.9]	200	83.7 [70.1–100.0]
	6.8 [5.8–8.0]	51.7	21.7 [18.1–25.9]
**3**	35.2 [31.7–39.0]	37.2 [30.9–44.9]	>200
	9.7 [8.8–10.8]	10.3 [8.5–12.4]	55.3
**4**	27.1 [22.5–32.7]	200	81.8 [75.1–89.0]
	7.5 [6.2–9.0]	55.3	22.6 [20.8–24.6]
**5**	18.0 [15.9–40.5]	100	76.9 [63.5–93.1]
	5.3 [4.7–11.9]	29.5	22.7 [18.7–27.4]
**7**	15.6 [14.0–16.9]	37.0 [26.9–50.9]	80.0 [71.5–89.5]
	4.1 [3.6–4.4]	9.6 [7.0–13.2]	20.8 [18.6–23.3]
**14**	28.4 [22.6–35.6]	100	100
	9.6 [7.6–12.0]	33.7	33.7
**28**	17.7 [15.6–20.0]	27.9 [22.2–35.0]	48.5 [42.1–50.9]
	8.8 [7.7–9.9]	13.8 [11.0–17.4]	21.4 [20.9–25.2]
**FLC**	255.9 [93.68–699]	0.04 [0.01–0.08]	1605 [549–4694]
	78.4 [28.7–214.1]	0.012 [0.003–0.025]	491 [168–1437]

^a,b^ IC_50_ value is defined as the concentration giving rise to an inhibition of growth of 50% compared to the drug-free control. Data are reported as mean values and 95% confident interval.

## Supplementary Materials

The following are available online. Table S1. Nonlinear regression parameters of the dose-response curves of chalcone **28** against *C. albicans* control strain and all *Candida* spp. clinical isolates; Figure S1. Dose-response curves of chalcone **28** obtained with non-*Candida* yeasts; Table S2. Nonlinear regression parameters of the dose-response curves of chalcone **28** against non *Candida* spp. clinical isolates.

## References

[1] Limper A.H., Adenis A., Le T., Harrison T.S. (2017). Fungal infections in HIV/AIDS. Lancet Infect. Dis..

[2] Brown G.D., Denning D.W., Gow N.A., Levitz S.M., Netea M.G., White T.C. (2012). Hidden killers: Human fungal infections. Sci. Transl. Med..

[3] Brown G.D., Denning D.W., Levitz S.M. (2012). Tackling human fungal infections. Science.

[4] Cavalheiro M., Teixeira M.C. (2018). Candida Biofilms: Threats, Challenges, and Promising Strategies. Front. Med..

[5] Favre S., Rougeron A., Levoir L., Perard B., Milpied N., Accoceberry I., Gabriel F., Vigouroux S. (2016). Saprochaete clavata invasive infection in a patient with severe aplastic anemia: Efficacy of voriconazole and liposomal amphotericin B with adjuvant granulocyte transfusions before neutrophil recovery following allogeneic bone marrow transplantation. Med. Mycol. Case Rep..

[6] Duran Graeff L., Seidel D., Vehreschild M.J., Hamprecht A., Kindo A., Racil Z., Demeter J., De Hoog S., Aurbach U., Ziegler M. (2017). Invasive infections due to Saprochaete and Geotrichum species: Report of 23 cases from the FungiScope Registry. Mycoses.

[7] Odds F.C., Brown A.J., Gow N.A. (2003). Antifungal agents: Mechanisms of action. Trends Microbiol..

[8] Sheng C., Zhang W. (2011). New lead structures in antifungal drug discovery. Curr. Med. Chem..

[9] Hamill R.J. (2013). Amphotericin B formulations: A comparative review of efficacy and toxicity. Drugs.

[10] Abreu A.C., McBain A.J., Simoes M. (2012). Plants as sources of new antimicrobials and resistance-modifying agents. Nat. Prod. Rep..

[11] Evans B.E., Rittle K.E., Bock M.G., DiPardo R.M., Freidinger R.M., Whitter W.L., Lundell G.F., Veber D.F., Anderson P.S., Chang R.S. (1988). Methods for drug discovery: Development of potent, selective, orally effective cholecystokinin antagonists. J. Med. Chem..

[12] Di Santo R. (2010). Natural products as antifungal agents against clinically relevant pathogens. Nat. Prod. Rep..

[13] Dimmock J.R., Elias D.W., Beazely M.A., Kandepu N.M. (1999). Bioactivities of chalcones. Curr. Med. Chem..

[14] Sahu N.K., Balbhadra S.S., Choudhary J., Kohli D.V. (2012). Exploring pharmacological significance of chalcone scaffold: A review. Curr. Med. Chem..

[15] Zhuang C., Zhang W., Sheng C., Zhang W., Xing C., Miao Z. (2017). Chalcone: A Privileged Structure in Medicinal Chemistry. Chem. Rev..

[16] Seleem D., Benso B., Noguti J., Pardi V., Murata R.M. (2016). In Vitro and In Vivo Antifungal Activity of Lichochalcone-A against Candida albicans Biofilms. PLoS ONE.

[17] Ortalli M., Ilari A., Colotti G., De Ionna I., Battista T., Bisi A., Gobbi S., Rampa A., Di Martino R.M.C., Gentilomi G.A. (2018). Identification of chalcone-based antileishmanial agents targeting trypanothione reductase. Eur. J. Med. Chem..

[18] Kishbaugh T.L.S. (2016). Pyridines and Imidazopyridines with Medicinal Significance. Curr. Top. Med. Chem..

[19] Hagmann W.K. (2008). The many roles for fluorine in medicinal chemistry. J. Med. Chem..

[20] Belluti F., De Simone A., Tarozzi A., Bartolini M., Djemil A., Bisi A., Gobbi S., Montanari S., Cavalli A., Andrisano V. (2014). Fluorinated benzophenone derivatives: Balanced multipotent agents for Alzheimer’s disease. Eur. J. Med. Chem..

[21] Rampa A., Tarozzi A., Mancini F., Pruccoli L., Di Martino R.M., Gobbi S., Bisi A., De Simone A., Palomba F., Zaccheroni N. (2016). Naturally Inspired Molecules as Multifunctional Agents for Alzheimer’s Disease Treatment. Molecules.

[22] Arendrup M.C., Cuenca-Estrella M., Lass-Florl C., Hope W., Eucast A. (2012). EUCAST technical note on the EUCAST definitive document EDef 7.2: Method for the determination of broth dilution minimum inhibitory concentrations of antifungal agents for yeasts EDef 7.2 (EUCAST-AFST). Clin. Microbiol. Infect..

[23] Zenger K., Dutta S., Wolff H., Genton M.G., Kraus B. (2015). In vitro structure-toxicity relationship of chalcones in human hepatic stellate cells. Toxicology.

[24] Cos P., Vlietinck A.J., Vanden Berghe D., Maes L. (2006). Anti-infective potential of natural products: How to develop a stronger in vitro ‘proof-of-concept’. J. Ethnopharmacol..

[25] Mahapatra D.K., Bharti S.K., Asati V. (2015). Chalcone scaffolds as anti-infective agents: Structural and molecular target perspectives. Eur. J. Med. Chem..

[26] Taff H.T., Mitchell K.F., Edward J.A., Andes D.R. (2013). Mechanisms of Candida biofilm drug resistance. Futur. Microbiol..

[27] Sardi J.C.O., Scorzoni L., Bernardi T., Fusco-Almeida A.M., Giannini M.J.S.M. (2013). Candida species: Current epidemiology, pathogenicity, biofilm formation, natural antifungal products and new therapeutic options. J. Med. Microb..

[28] Bonvicini F., Antognoni F., Iannello C., Maxia A., Poli F., Gentilomi G.A. (2014). Relevant and selective activity of Pancratium illyricum L. against Candida albicans clinical isolates: A combined effect on yeast growth and virulence. BMC Complement. Altern. Med..

[29] Sadowska B., Budzynska A., Stochmal A., Zuchowski J., Rozalska B. (2017). Novel properties of Hippophae rhamnoides L. twig and leaf extracts - anti-virulence action and synergy with antifungals studied in vitro on Candida spp. model. Microb. Pathog..

[30] Melo A.S., Bizerra F.C., Freymuller E., Arthington-Skaggs B.A., Colombo A.L. (2011). Biofilm production and evaluation of antifungal susceptibility amongst clinical Candida spp. isolates, including strains of the Candida parapsilosis complex. Med. Mycol..

