# Supplementary material for: Functionalization of the Chalcone Scaffold for the Discovery of Novel Lead Compounds Targeting Fungal Infections
Source: Molecules. 2019 Jan 21;24(2):372. doi: 10.3390/molecules24020372 (PMC6359675; doi:10.3390/molecules24020372)
Supplement: Supplementary file 1 [file molecules-24-00372-s001.pdf]

Article

Functionalization of the chalcone scaffold for the discovery of novel lead compounds targeting fungal infections

Francesca Bonvicini[1], Giovanna A. Gentilomi[1,2], Francesca Bressan[3], Silvia Gobbi[3], Angela Rampa[3], Alessandra Bisi[3], and Federica Belluti[3]*

1 Department of Pharmacy and Biotechnology, Alma Mater Studiorum University of Bologna, Via Massarenti 9, 40138 Bologna, Italy

2 Unit of Microbiology, Alma Mater Studiorum-University of Bologna, S. Orsola-Malpighi Hospital, Via Massarenti 9,40138 Bologna, Italy

3 Department of Pharmacy and Biotechnology, Alma Mater Studiorum-University of Bologna, Via Belmeloro 6, 40126 Bologna, Italy

* Correspondence: federica.belluti@unibo.it, tel +39 0512099732 (FB).

Table S1. Nonlinear regression parameters of the dose–response curves of chalcone **28** against *C. albicans* control strain and all *Candida* spp. clinical isolates.

	C. albicans ATCC 10231	***C. albicans***	***C. tropicalis***	***C. krusei***
log(inhibitor) vs. normalized response -- Variable slope				
Best-fit values				
LogIC50	1745	1682	1424	1804
HillSlope	5619	2587	4630	3811
IC50	55.63	48.08	26.55	63.64
Std. Error				
LogIC50	0.01302	0.02046	0.01909	0.01413
HillSlope	1187	0.2945	1270	0.3731
95% Confidence Intervals				
LogIC50	1.719 to 1.772	1.640 to 1.724	1.385 to 1.463	1.775 to 1.833
HillSlope	3.188 to 8.050	1.979 to 3.195	2.014 to 7.245	3.041 to 4.581
IC50	52.32 to 59.15	43.62 to 52.99	24.25 to 29.07	59.50 to 68.06
Goodness of Fit				
Degrees of Freedom	28	24	25	24
R^2	0.9718	0.9357	0.9242	0.9648
Absolute Sum of Squares	1463	1992	3635	1269
Sy.x	7229	9110	12.06	7272
Number of points				
Analyzed	30	26	27	26

	C. parapsilosis	***C. lusitaniae***	***C. utilis***	***C. glabrata***

log(inhibitor) vs. normalized response -- Variable slope				
Best-fit values				
LogIC$_{50}$	1496	1423	1471	1555
HillSlope	1207	1731	8667	3339
IC50	31.33	26.49	29.57	35.93
Std. Error				
LogIC$_{50}$	0.03112	0.05841	0.01256	0.02515
HillSlope	0.1251	0.4021	1463	0.5094
95% Confidence Intervals				
LogIC$_{50}$	1.432 to 1.560	1.302 to 1.544	1.445 to 1.497	1.504 to 1.607
HillSlope	0.9484 to 1.466	0.9010 to 2.561	5.654 to 11.68	2.288 to 4.391
IC$_{50}$	27.01 to 36.34	20.07 to 34.96	27.86 to 31.39	31.88 to 40.49
Goodness of Fit				
Degrees of Freedom	23	24	25	24
R^2	0.8982	0.6885	0.9969	0.9071
Absolute Sum of Squares	1723	10,359	164.5	3658
Sy.x	8654	20.78	2565	12.35
Number of points				
Analyzed	25	26	27	26

Figure S1. Dose–response curves of chalcone **28** obtained with non-*Candida* yeasts.

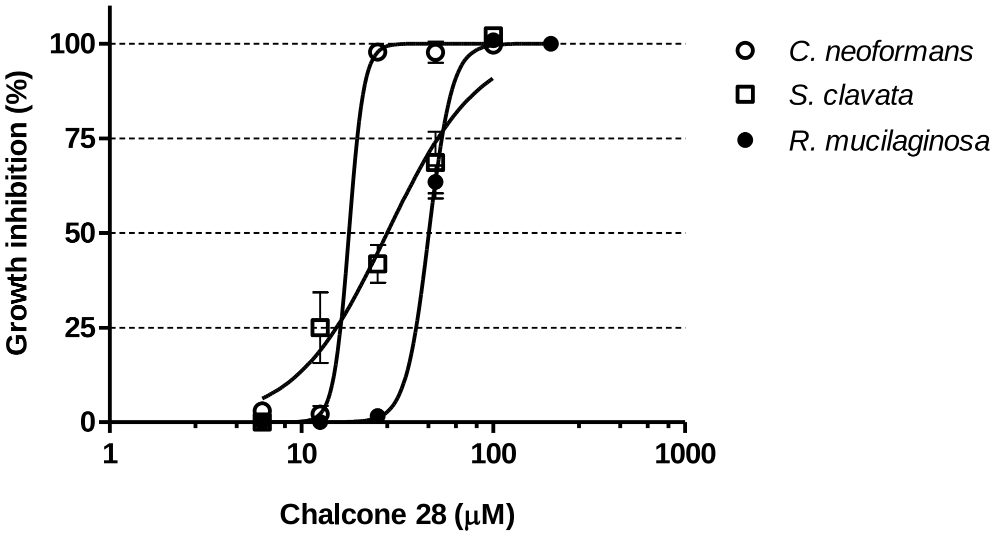

Table S2. Nonlinear regression parameters of the dose–response curves of chalcone **28** against non-*Candida* spp. clinical isolates.

	C. neoformans	*S. clavata*	*R. mucilaginosa*
**log(inhibitor) vs. normalized response -- Variable slope**			
**Best-fit values**			
**LogIC50**	1248	1447	1665
**HillSlope**	10.99	1806	7134
**IC50**	17.68	28.01	46.25
**Std. Error**			
**LogIC50**	0.02614	0.04636	0.01277
**HillSlope**	1909	0.3423	2463
**95% Confidence Intervals**			
**LogIC50**	1.193 to 1.302	1.351 to 1.543	1.639 to 1.692
**HillSlope**	7.034 to 14.95	1.097 to 2.514	2.010 to 12.26
**IC50**	15.61 to 20.03	22.46 to 34.93	43.51 to 49.17
**Goodness of Fit**			
**Degrees of Freedom**	22	23	21
R^2	0.9917	0.7732	0.9863
**Absolute Sum of Squares**	345.8	6606	597.6
**Sy.x**	3964	16.95	5335
**Number of points**			
**Analyzed**	24	25	23
